# High-Throughput Phenotypic Characterization of *Pseudomonas aeruginosa* Membrane Transport Genes

**DOI:** 10.1371/journal.pgen.1000211

**Published:** 2008-10-03

**Authors:** Daniel A. Johnson, Sasha G. Tetu, Katherine Phillippy, Joan Chen, Qinghu Ren, Ian T. Paulsen

**Affiliations:** 1 J. Craig Venter Institute, Rockville, Maryland, United States of America; 2 Macquarie University, Sydney, New South Wales, Australia; Progentech, United States of America

## Abstract

The deluge of data generated by genome sequencing has led to an increasing reliance on bioinformatic predictions, since the traditional experimental approach of characterizing gene function one at a time cannot possibly keep pace with the sequence-based discovery of novel genes. We have utilized Biolog phenotype MicroArrays to identify phenotypes of gene knockout mutants in the opportunistic pathogen and versatile soil bacterium *Pseudomonas aeruginosa* in a relatively high-throughput fashion. Seventy-eight *P. aeruginosa* mutants defective in predicted sugar and amino acid membrane transporter genes were screened and clear phenotypes were identified for 27 of these. In all cases, these phenotypes were confirmed by independent growth assays on minimal media. Using qRT-PCR, we demonstrate that the expression levels of 11 of these transporter genes were induced from 4- to 90-fold by their substrates identified via phenotype analysis. Overall, the experimental data showed the bioinformatic predictions to be largely correct in 22 out of 27 cases, and led to the identification of novel transporter genes and a potentially new histamine catabolic pathway. Thus, rapid phenotype identification assays are an invaluable tool for confirming and extending bioinformatic predictions.

## Author Summary

Genome sequencing has led to the identification of literally millions of new genes, for which there is no experimental evidence concerning their function. This limits our knowledge of these genes to computational predictions; however, the accuracy of such bioinformatic predictions is essentially unknown. We have focused on investigating the accuracy of bioinformatic predictions for a specific class of genes—those encoding membrane transporters. Our approach used Biolog phenotype MicroArrays to screen transporter gene knockout mutants in the bacterium *P. aeruginosa* for the ability to metabolize hundreds of different compounds. We were able to identify functions for 27 out of 78 genes, all of which were confirmed through independent growth assays. For 80% of these genes, the computationally predicted and experimentally determined functions were either identical or generically similar. Additionally, this led to the discovery of entirely new types of transporters and a novel potential histamine metabolic pathway.

## Introduction

The genomic era has provided us with hundreds of complete microbial genome sequences, and has allowed us to generate genome sequences from whole environments using a “metagenomics” approach [1],[2]. Taken together, the sequencing of individual genomes and whole communities has enabled the realization of a level of genetic diversity and complexity that was previously unappreciated. This massive volume of data has led to an increasing reliance on bioinformatic predictions, since the traditional experimental approach of characterizing gene function one at a time can not keep pace with the sequence-based discovery of novel putative genes. Automated bioinformatic pipelines together with manual curation by expert human annotators typically allow the functional predictions for 50–70% of the genes of a newly sequenced microorganism [3],[4].

Bioinformatic predictions are largely based on sequence similarity to known proteins based on BLAST, Hidden Markov Model or other searches, along with supporting evidence based on genome context methods [5]. *Ab initio* prediction methods remain highly speculative in the absence of other evidence, hence bioinformatic gene function predictions are essentially limited to what we already know experimentally from other systems. Furthermore, the accuracy of bioinformatic predictions remains largely undetermined, i.e., the likelihood of any single gene functional assignment being correct is at best an educated guess.

Our group has focused on bioinformatic predictions of membrane transporter function, developing a pipeline for annotation of membrane transport genes and a relational database, TransportDB, describing the predicted transporter content of all sequenced genomes [6],[7]. Hence we have been interested in finding approaches to functionally characterize transporter genes in a high throughput fashion to assess the accuracy of our bioinformatic predictions. In some aspects, membrane transport genes are good candidates for high throughput phenotypic screens, since in many cases individual knockout mutants might be expected to give relatively simple phenotypes, e.g., loss of a glucose transporter causing a defect on growth on glucose as a sole carbon source. Of course, the presence of multiple transporters with overlapping specificities, indirect effects from loss of a transporter and other phenomenon, have the capacity to complicate such a simplistic scenario and must be taken into account when analyzing data.

One technology that has the potential to accelerate the functional characterization of genes is Biolog phenotype MicroArrays, a respiration-based assay system that can test up to 2000 phenotypic traits simultaneously [8]. This system uses 96 well plates where each well tests a separate phenotype using a tetrazolium redox dye that produces a color change in response to cellular respiration. The detection system is a Biolog OmniLog incubator/reader that cycles each plate in front of an imaging head every 15 minutes, measuring and recording the color change from reduction of the tetrazolium dye in each well, providing a quantitative kinetic plot of color formation against time. This technology has been used previously to facilitate both characterization of transporters [9]–[11] and the testing of bioinformatic predictions [12],[13].

In this study, we have focused on characterizing a collection of knockout mutants of integral cytoplasmic membrane transporter genes in the ecologically and metabolically diverse bacterium *Pseudomonas aeruginosa* using Biolog phenotype MicroArrays in conjunction with more traditional experimental approaches. *P. aeruginosa* is known to be capable of growth on a broad range of substrates such as amino acids, carboxylates, aromatic compounds, but on only a narrow selection of carbohydrates [14]. Consistent with this, more than 300 predicted cytoplasmic membrane transport systems were identified in *P. aeruginosa* in the initial characterization of its genome [15].

## Results

### Transporter Annotation Update

One of the objectives of this study was to systematically compare the bioinformatic predictions of transporter function with the high throughput functional characterization of transporters. To provide a good basis for these comparisons we decided to update the original annotation of the *P. aeruginosa* membrane transporters (more than seven years has passed since the original genome annotation was performed [15]), as well as undertake a subjective estimate of our bioinformatic annotation.

First, the complete *P. aeruginosa* gene set was run through our TransportDB automated annotation pipeline [6]. Second, all of the transporters identified by this approach or by the original genome annotation were analyzed phylogenetically to investigate their evolutionary histories and to determine whether or not they could clearly be identified as orthologues of known membrane transporters. Third, we analyzed their comparative genome context, looking at their flanking genes in *P. aeruginosa*, but also examining the flanking genes of homologous transporters encoded in other genomes.

Based on these analyses, a total of 427 predicted membrane transport genes were identified and assigned functions and family groupings (Table S1). Using this approach, 124 “hypothetical proteins” from the original *P. aeruginosa* PA01 genome annotation [15], were annotated as membrane transporters with specific substrates or general functions. These include newly identified paralogues in major transporter families such as ATP binding cassette (ABC), major facilitator superfamily (MFS), and drug/metabolite transporter (DMT) superfamily. In addition, our analyses detected transporters belonging to several new transporter families that were identified after the genome annotation was published, for example, the tricarboxylate transporter (TTT) family [16] and the aromatic acid exporter (ArAE) family [9].

One of the key objectives of this study was to compare bioinformatic predictions with high throughput functional data, in order to obtain an estimate of the accuracy of our bioinformatic predictions. Based on current knowledge of transporter systems, of the 427 predicted transporters, 16 (4%) were previously experimentally characterized transporters in *P. aeruginosa*, 116 (27%) were clear orthologues to experimentally characterized transporters from other species, such as *E. coli*, and other *Pseudomonas* species, 229 (54%) were transporters whose specificities were predicted with high bioinformatic confidence, while the remaining 66 (15%) had weak bioinformatic evidence supporting their function as transporters.

Additionally we compared the transporter complement of *P. aeruginosa* PA01 with the recently sequenced clinical isolate *P. aeruginosa* UCBPP-PA14 [17]. These two strains are very similar in their overall transporter complement with the most notable differences being in respect to additional iron and nickel uptake systems present in the clinical PA14 strain. There was almost no difference in terms of predicted uptake capabilities for amino acids and sugars. The complete list of predicted PA14 transporters from this analysis is available on www.membranetransport.org.

### Biolog Phenotype MicroArray Analysis

We obtained a subset of 384 *P. aeruginosa* transporter gene knockout mutants from a larger collection of *P. aeruginosa* Mini-Tn*5*-Tc^r^ gene knockout mutants. This collection of mutants was constructed in two different genetic backgrounds. The isogenic parent of the majority of the Tn5 knockouts was strain PAK, the parent of the remainder of these was the sequenced strain PA01 [15],[18]. Knockout mutants from the larger collection have also been used in studies by other research groups [19],[20].

Biolog phenotype MicroArrays were employed to identify phenotypes for a subset of the transporter gene mutants. In this initial study we chose to focus on characterizing the subset of knockout mutants which were predicted to encode amino acid and sugar phosphotransferase system (PTS) transporters. Amino acid transporters were a focus as there is very good coverage of amino acids and derivatives as carbon and/or nitrogen sources on the Biolog phenotype MicroArrays. Also, previous experimental studies have described arginine/ornithine [21] and branched chain amino acid transporters [22] in *P. aeruginosa*. The (PTS) transporter mutants were chosen for further analysis as the bioinformatic predictions for these tended to have a high degree of confidence, in contrast to the amino acid transporters.

Based on the updated annotation, our mutant collection included 78 knockout mutants of putative amino acid transporter or sugar PTS genes (Table 1). This included 46 transporters with a precise specificity and 32 transporters with a more generic prediction. The transposon insertion site of each of these mutants had been sequenced by PathoGenesis Corporation. We additionally confirmed the identities of each of these mutants by PCR with primers specific to each gene and the transposon insert (data not shown).

**Table 1:** Details of knockout mutants of *Pseudomonas aeruginosa* putative transporter genes.

Gene	Host Strain	Predicted Function
PA0129	PAK	4-amino butyrate APC family transporter (*gabP*)
PA0220	PAK	amino acid APC family transporter
PA0313	PAK	amino acid ABC transporter membrane protein
PA0314	PAK	amino acid ABC transporter periplasmic binding protein
PA0322	PAK	cationic amino acid APC family transporter
PA0783	PAK	proline/sodium transporter
PA0789	PAK	proline APC family transporter
PA0866	PAK	aromatic amino acid APC family transporter
PA0888	PAK pili-	arginine/ornithine ABC transporter periplasmic binding protein (*AotJ*)
PA0889	PA01	arginine/ornithine ABC transporter membrane protein (*AotQ*)
PA0890	PAK	arginine/ornithine ABC transporter membrane protein (*AotM*)
PA0892	PAK	arginine/ornithine ABC transporter ATP binding protein (*AotP*)
PA1070	PAK	branched chain amino acid ABC transporter ATP binding protein (*BraG*)
PA1071	PAK	branched chain amino acid ABC transporter ATP binding protein (*BraF*)
PA1072	PAK	branched chain amino acid ABC transporter membrane protein (*BraE*)
PA1073	PAK	branched chain amino acid ABC transporter membrane protein (*BraD*)
PA1074	PAK	branched chain amino acid ABC transporter periplasmic binding protein (*BraC*)
PA1147	PAK	amino acid APC family transporter
PA1194	PAK	arginine/ornithine APC family transporter
PA1256	PAK	amino acid ABC transporter ATP binding protein
PA1257	PA01	amino acid ABC transporter membrane protein
PA1258	PAK	amino acid ABC transporter membrane protein
PA1260	PAK	amino acid ABC transporter periplasmic binding protein
PA1339	PAK	amino acid ABC transporter ATP binding protein
PA1340	PAK	amino acid ABC transporter membrane protein
PA1341	PAK	amino acid ABC transporter membrane protein
PA1418	PAK	sodium∶solute symporter
PA1485	PA01	amino acid APC family transporter
PA1590	PAK	branched chain amino acid sodium ion symporter (*BraB*)
PA1819	PAK	amino acid APC family transporter
PA1958	PAK	nicotinamide mononucleotide transporter
PA1971	PA01	branched chain amino acid∶cation symporter (*BraZ*)
PA2041	PAK	amino acid APC family transporter
PA2079	PA01	amino acid APC family transporter
PA2202	PAK	amino acid ABC transporter membrane protein
PA2203	PAK	amino acid ABC transporter membrane protein
PA2204	PA01	amino acid ABC transporter periplasmic binding protein
PA2252	PA01	alanine/glycine/sodium symporter
PA2307	PAK	nitrate/sulfonate/taurine ABC transporter membrane protein
PA2533	PAK	alanine/glycine/sodium symporter
PA2923	PAK	histidine ABC transporter periplasmic binding protein (*HisJ*)
PA2924	PAK	histidine ABC transporter membrane protein (*HisQ*)
PA2925	PAK	histidine ABC transporter membrane protein (*HisM*)
PA2926	PAK	histidine ABC transporter ATP binding protein (*HisP*)
PA3000	PAK	aromatic amino acid APC family transporter (*AroP1*)
PA3176	PA01	glutamate/sodium ion symporter (*GltS*)
PA3560	PAK	phosphotransferase system transporter fructose-specific IIBC component (*FruA*)
PA3562	PAK	phosphotransferase system transporter enzyme I (*FruI*)
PA3597	PAK	amino acid APC family transporter
PA3641	PAK	alanine/sodium symporter
PA3760	PAK	N-Acetyl-D-Glucosamine acphosphotransferase system transporter
PA3761	PAK	N-Acetyl-D-Glucosamine phosphotransferase system transporter
PA3865	PAK	arginine/ornithine ABC transporter periplasmic binding protein
PA3889	PAK	glycine ABC transporter periplasmic binding protein
PA4023	PAK	ethanolamine APC family transporter
PA4072	PAK	amino acid APC family transporter
PA4096	PAK	MFS transporter
PA4192	PAK	amino acid ABC transporter ATP binding protein
PA4193	PAK	amino acid ABC transporter membrane protein
PA4194	PAK	amino acid ABC transporter membrane protein
PA4195	PAK	amino acid ABC transporter periplasmic binding protein
PA4233	PA01	MFS transporter
PA4628	PAK	lysine APC family transporter
PA4804	PAK	amino acid APC family transporter
PA4909	PA01	branched chain amino acid ABC transporter ATP binding protein
PA4910	PAK	branched chain amino acid ABC transporter ATP binding protein
PA4911	PA01	branched chain amino acid ABC transporter membrane protein
PA4912	PAK	branched chain amino acid ABC transporter membrane protein
PA4981	PA01	amino acid APC family transporter
PA5074	PAK	glutamine ABC transporter ATP binding protein
PA5075	PA01	glutamine ABC transporter membrane protein
PA5076	PAK	glutamine ABC transporter periplasmic binding protein
PA5097	PAK	proline APC family transporter
PA5153	PAK	amino acid (lysine/arginine/ornithine/histidine/octopine) ABC transporter periplasmic binding protein
PA5155	PAK	amino acid (lysine/arginine/ornithine/histidine/octopine) ABC transporter membrane protein
PA5170	PAK	arginine/ornithine APC family antiporter (*ArcD*)
PA5504	PA01	D-methionine ABC transporter membrane protein
PA5510	PAK	amino acid APC family transporter

**Figure 1:**
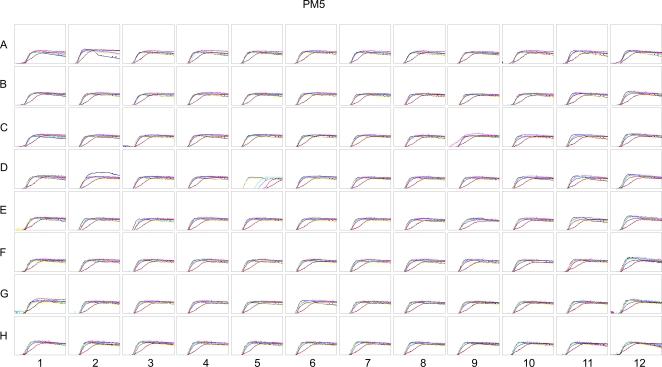
Reproducibility of Biolog Phenotype MicroArray data with wild type *P. aeruginosa* strains, PA01, and PAK. An overlay of data from plate PM5, representing multiple replicates of assays with each of the parental strains PAK and PAO1, illustrates the degree of reproducibility possible with the plate assay system. Note that for most substrates there is minimal variability on the PM5 plate, with the exception of well D5, containing the substrate homoserine lactone.

We initially optimized conditions for utilizing phenotype MicroArrays with *P. aeruginosa*, including modifying the initial growth medium to diminish production of pigmented compounds by the bacterium that interfered with recording of the colourimetric reaction in each plate by the Biolog OmniLog incubator/reader. Reproducibility of the *P. aeruginosa* PM data was investigated by running multiple replicates of each of the parental strains PAK and PAO1 on separate days. Figure 1 displays an overlay of multiple experiments for these strains on the PM5 plate, where it can be seen that, overall, there is a fairly high level of reproducibility. However, reproducibility did vary somewhat between specific wells/compounds, for example, well D5 on the PM5 plate, containing homoserine lactone, showed a substantially higher level of variation than other wells on the same plate (Figure 1). A comparison of the substrate utilization capabilities of the two different background strains, PAK and PAO1 indicated that these two strains had highly comparable utilization patterns that differed primarily in terms of different dipeptides.

**Figure 2:**
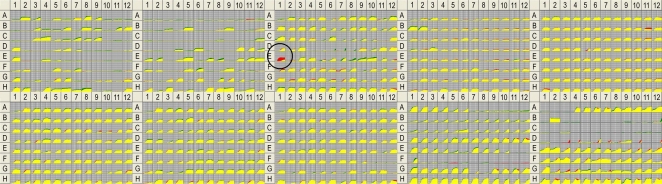
Data for Biolog Phenotype MicroArray PM1-10 plates comparing one mutant (PA0220 knockout) with its isogenic parent, PAK. This mutant shows evidence of a significant defect in histamine utilization (well E1 on PM3 plate, circled). Other minor differences in substrate utilization may be noted in this example (reduced utilization of the mutant compared to parent strain indicated by red areas in the growth curve for each substrate, increased utilization in the mutant by green areas).

The 78 predicted amino acid or carbohydrate transporters were phenotypically screened on PM1-10 plates and compared with their isogenic parental strains. An example of the data generated via the Biolog OmniLog reader is shown in Figure 2, which compares a mutant (PA0220 knockout) with its isogenic parent, PAK, on PM1-10 plates, showing evidence of a significant defect in histamine utilization (well E1 on PM3 plate). Other minor differences in substrate utilization may be noted in this example, whose significance is not readily apparent (Figure 2).

**Figure 3:**
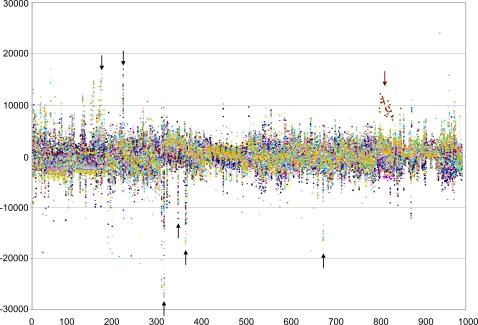
Graphical display of substrate utilization data from Biolog plates PM1-10 for all mutant and parent strain comparisons. Each point on the horizontal axis represents a particular well on a particular plate, the vertical axis represents the area difference (in arbitrary units) under the kinetic curve of dye formation between the mutant and wild type over a 48 hour time period (see Materials and Methods). The black arrows highlight some examples of wells where substrate utilization showed a high degree of variability across the strain tested. The method of pooled analysis used here draws attention to anomalous data, highlighting potentially problematic plates or substrates, which may require further confirmatory assays. The brown arrow highlights the outlier data points produced by a faulty plate, retesting produced more typical data for this mutant.

In order to minimize problems arising from the higher variability in some substrate wells, and to make use of the large dataset, the data from all 78 mutant and parent strain comparisons were analyzed concurrently (Figure 3). This facilitated identification of statistically significant phenotypic differences. A statistical analysis of this combined data identified phenotypes that showed substantial defects in substrate utilization compared to the parental strain, differing from the mean by more than two standard deviations. Based on this criterion we were able to assign significant phenotypes to 27 of the 78 genes (35%) tested (Table 2).

**Table 2:** Phenotypes of *P. aeruginosa* transport mutants, based on Biolog phenotype MicroArray and minimal media growth assays.

Gene	Predicted function	Biolog Phenotypes^a^	Plate growth^b^
PA0220	amino acid APC family transporter	Histamine – N	-
PA0783	proline/sodium transporter	L-Proline - C+N	-
PA0888	arginine/ornithine ABC transporter periplasmic binding protein (*AotJ*)	L-Arginine – C	-
		L-Ornithine – C	-
PA0889	arginine/ornithine ABC transporter membrane protein (*AotQ*)	L-Arginine – C	+/−
		L-Ornithine – C	+/−
PA0890	arginine/ornithine ABC transporter membrane protein (*AotM*)	L-Arginine – C	-
		L-Ornithine – C	-
PA0892	arginine/ornithine ABC transporter ATP binding protein (*AotP*)	L-Arginine – C	-
		L-Ornithine – C	-
PA1070	branched chain amino acid ABC transporter ATP binding protein (*BraG*)	D-Alanine – C	+/−
		L-Alanine – C	+/−
PA1071	branched chain amino acid ABC transporter ATP binding protein (*BraF*)	D-Alanine – C	+/−
		L-Alanine – C	+/−
		L-Isoleucine – C	+/−
		L-Valine – N	+/−
		d-Amino Valeric Acid – C	+/−
PA1072	branched chain amino acid ABC transporter membrane protein (*BraE*)	D-Alanine – C	+/−
PA1073	branched chain amino acid ABC transporter membrane protein (*BraD*)	D-Alanine – C	+/−
		L-Alanine – C	-
		L-Isoleucine – C	+/−
PA1074	branched chain amino acid ABC transporter periplasmic binding protein (*BraC*)	D-Alanine – C	+/−
		L-Alanine – C	-
		L-Isoleucine – C	+/−
		L-Valine – N	-
PA1256	amino acid ABC transporter ATP binding protein	Hydroxy-L-Proline -C	+/−
PA1257	amino acid ABC transporter membrane protein	Hydroxy-L-Proline -C	-
PA1260	amino acid ABC transporter periplasmic binding protein	Hydroxy-L-Proline -C	+/−
PA1339	amino acid ABC transporter ATP binding protein	L-Glutamic Acid - C	+/−
PA1340	amino acid ABC transporter membrane protein	L-Glutamic Acid - C	+/−
		L-Glutamine – C	+/−
		L-Glutamic Acid - N	+/−
		D-Glutamic Acid - N	-
		N-Acetyl-L-Glutamic Acid – N	+/−
PA1341	amino acid ABC transporter membrane protein	L-Glutamic Acid -C	+/−
PA3176	glutamate/sodium ion symporter (*GltS*)	N-Acetyl-L-Glutamic Acid - N	+/−
PA3560	phosphotransferase system transporter fructose-specific IIBC component (*FruA*)	D-Fructose – C	-
PA3562	phosphotransferase system transporter enzyme I (*FruI*)	D-Fructose – C	-
PA3760	N-Acetyl-D-Glucosamine phosphotransferase system transporter	N-Acetyl-D-Glucosamine – N	-
PA3761	N-Acetyl-D-Glucosamine phosphotransferase system transporter	N-Acetyl-D-Glucosamine – C+N	-
PA4910	branched chain amino acid ABC transporter ATP binding protein	D-Alanine – N	+/−
PA4912	branched chain amino acid ABC transporter membrane protein	D-Alanine – N	+/−
		D-Valine – N	+/−
PA5153	amino acid (lysine/arginine/ornithine/histidine/octopine) ABC transporter periplasmic binding protein	L-Ornithine – C	+/−
PA5155	amino acid (lysine/arginine/ornithine/histidine/octopine) ABC transporter membrane protein	L-Ornithine – C	-
PA5504	D-methionine ABC transporter membrane protein	L-Histidine – C	+/−

a Knockout mutants with a significant reduction in substrate utilization as assayed by Biolog phenotype MicroArray plates. Substrates used as carbon sources are labeled with C, substrates used as nitrogen sources are labeled with N.

b Growth on minimal media containing specified sole carbon or nitrogen source, - indicates no growth or pinpoint colonies of mutant, +/− indicates growth of mutant was reduced compared to wild type.

This pooled analysis allows the amount of noise in the data to be gauged more accurately (as visualized in Figure 3 by the central band extending from approximately −5000 to 5000 depending on the particular well/plate) and highlights substrates which show systematic variability. For instance, the wells that show extended vertical lines (examples include, PM4, well E8 methylene diphosphonic acid; PM4, well A7, hypophosphite; PM3, well A8, L-cysteine) represent wells that show greater variability and whose data is probably not reliable for any individual mutant. Another benefit of this type of analysis is that anomalous results are easily detected, such as in one instance where a particular plate was defective for one set of experiments (see the cluster of brown dots highlighted above the noise band in Figure 3, a repeat of this experiment produced a more typical result).

### Confirmatory Assays

Independent growth studies on minimal media were undertaken to confirm the phenotypes of the mutants with discernable phenotypes from the Biolog PM screen. Growth data for each mutant in minimal media with the appropriate carbon and nitrogen sources are shown in Table 2. These growth assays confirmed the phenotypes of 27 mutants, with some minor discrepancies with regard to the ability of specific substrates to act as both sole carbon and nitrogen source.

Expression of many transporter genes is regulated in response to their transported substrate [23]. The ability of the putative substrates of each of the 27 transporter genes to induce expression of the respective genes in *P. aeruginosa* PAK was examined by qRT-PCR. Expression of 11 out of the 27 genes was induced by between 5- to 86-fold by their transporter substrates (Table 3). Expression of the other 17 genes was observed, but appeared to be constitutive under the conditions tested.

**Table 3:** Putative transporter mutants found to be induced in qRT-PCR experiments.

Gene	qRT-PCR inducer^a^	Ratio^b^
PA0220	Histamine	35.8
PA0783	Proline	7.0
PA1256	Hydroxy-L-Proline	86.3
PA1257	Hydroxy-L-Proline	15.5
PA1260	Hydroxy-L-Proline	43.9
PA1339	L-Glutamic Acid	8.0
PA1340	L-Glutamic Acid	9.4
PA3560	D-Fructose	4.3
PA3562	D-Fructose	27.1
PA3760	N-Acetyl-D-Glucosamine	5.2
PA3761	N-Acetyl-D-Glucosamine	5.1

a The compounds used to induce expression were the probable transported substrates as determined by Biolog phenotype and plate growth assays.

b The ratio of gene expression for each knockout mutant compared to its isogenic parent strain, under induced conditions. The differences were significant, with p-values of <0.05 recorded for each (the highest was 0.016).

### Transporter Functions

There are two clusters of sugar PTS genes encoded within the *P. aeruginosa* PAO1 genome [24]. The first of these, PA3560 and PA3562 encode a putative fructose enzyme IIBC and a fused putative enzyme I/HPr/enzyme IIA fructose. These are encoded in a putative operon with PA3561, a predicted 1-phosphofructokinase. Knockouts in either PA3560 or PA3562 were defective in growth on D-fructose as a sole carbon source, based on both Biolog and growth data (Table 2). Expression of both of these genes is induced by D-fructose, supporting the notion that these genes form a dedicated, inducible system for fructose uptake (Table 3).

The second cluster of PTS genes encodes a putative fused enzyme I/HPr/enzyme IIA and a putative enzyme IIBC. These two genes are located at the end of a putative operon encoding glucosamine utilization genes, suggesting N-acetylglucosamine as a potential substrate for the PTS transporter. Knockouts in either PA3760 or PA3761 were defective in growth on N-acetylglucosamine as a sole carbon source, based on both Biolog and growth data (Table 2). As for the fructose PTS genes, expression of the N-acetylglucosamine PTS genes was induced by their apparent transported substrate (Table 3).

We were able to identify specific phenotypes for eight predicted amino acid transporter genes that had only generalized bioinformatic predictions of function. PA5504 encodes a predicted membrane component of an ABC amino acid transporter, and is encoded in a putative two gene operon along with an ATP-binding component gene. A PA5504 knockout affected growth on L-histidine as a carbon source (Table 2), however its expression was not induced by this compound. A noninducible histidine outer membrane porin OpdC has been previously identified [20]. However, since it is encoded in a different region of the genome, the interdependence of these two systems is unclear.

PA1256, PA1257 and PA1260 are all components of a predicted ABC amino acid transporter encoded in a putative operon along with two hypothetical genes. Knockouts in these genes affected growth on hydroxy-L-proline as a carbon source, and expression of all three genes was induced by hydroxy-L-proline (Table 2 and 3). There was no growth defect on L-proline, nor was expression affected by L-proline as an inducer, suggesting specificity for hydroxy-L-proline. Although annotated as a hypothetical protein, the product of PA1255 showed sequence similarity to proline racemases. To our knowledge these would be the first genes identified for a hydroxy-L-proline transporter.

PA1339, PA1340 and PA1341 all encode components of a predicted ABC amino acid transporter, and are located in a putative three gene operon. All three mutants were defective for utilization of L-glutamic acid as a carbon source, and expression of two of these genes was induced by L-glutamic acid (Table 2 and 3). Additionally, one of these mutants (PA1340) also showed significantly decreased utilization of the related compounds, L-glutamine, D-glutamic acid, and N-acetyl-L-glutamic acid. Examination of the Biolog data indicated that the other two mutants showed reduced utilization of these three compounds that was below the two standard deviation significance cutoff employed, suggesting that this transport system is probably specific for all four of these substrates.

The last predicted amino acid transporter with a generic specificity was PA0220, and disruption of this gene affected utilization of histamine as a nitrogen source. Expression of PA0220 was strongly induced by histamine (Table 3) and as discussed below may be co-encoded with two genes that potentially form a novel histamine utilization pathway. This is particularly interesting as no transporter for histamine has been described at a molecular level previously.

Fifteen mutants were phenotypically characterized for which precise substrate specificity predictions had been made. PA0783, located in a putative monocistronic operon, was predicted to encode a proline uptake system, and a knockout mutant affected utilization of L-proline as both a carbon and nitrogen source (Table 2). PA0783 also displayed L-proline-inducible expression (Table 3).

The predicted glutamate transporter gene PA3176 is the last gene in a three gene operon, also encoding a putative regulator and a homologue of formiminoglutamate hydrolase (PA3175). Disruption of PA3176 had no effect on utilization of glutamate but did disrupt utilization of N-acetyl-L-glutamate (Table 2). This presents the possibility that the role of PA3175 is to cleave off the acetyl group to yield L-glutamate. These two genes may therefore represent a novel transporter and catabolic enzyme for utilization of N-acetyl-L-glutamate.

Genes PA4910 and PA4912 comprise two genes of a five gene operon predicted to be involved in transport of branched chain amino acids. PA4910 encodes a putative ATP-binding protein, while PA4912 encodes a predicted membrane component of this ABC amino acid transporter. Disruption of PA4910 resulted in reduced utilization of D-Alanine (as a nitrogen source), while PA4912 mutants had disrupted D-Alanine and D-Valine utilization (Table 2).

PA5153 and PA5155 are two genes of a four gene operon proposed to encode an arginine/ornithine transport system. Growth experiments indicated that both these mutants showed reduced growth on L-Ornithine. This operon has previously been shown to be arginine-inducible under control of the argR regulator [25], however under our experimental conditions using qRT-PCR we did not observe any induction by arginine.

Genes PA0888, PA0889, PA0890 and PA0892, comprise four of the six genes which make up the *aot* operon (*aotJ*, *aotQ*, *aotM* and *aotP*, respectively), previously shown to be involved in arginine/ornithine uptake [21]. PA0888 is thought to encode an arginine/ornithine transport system substrate binding protein, PA0889 and PA0890 encode putative permease proteins while PA0892 is predicted to encode the associated ATP-binding component gene. Disruption of each of these four genes resulted in mutants defective in arginine/ornithine uptake, as expected (Table 2).

PA1070, PA1071, PA1072, PA1073, PA1074, which correspond to *braG*, *braF*, *braE*, *braD* and *braC*, comprise a five gene operon which has also been characterized experimentally. The *braC* has been shown to encode the binding protein for branched-chain amino acids [26], *braF* and *braG* genes are thought to encode ATP-binding proteins, while *braE* and *braD* encode very hydrophobic proteins [22]. Complementation experiments have shown that each of these genes is essential for correct functioning of the high affinity branched-chain amino acid transport system encoded by the *bra* operon [27]. In this work, mutants for each of these genes were found to be defective in Alanine metabolism, while utilization of L-Valine and L-Isoleucine was also altered in some cases (PA1074 and PA1071 were also defective in d-Amino Valeric Acid uptake, Table 2).

### Mapping Phenotype MicroArray Data onto PseudoCyc

The 27 transporter genes with confirmed phenotypes appear to be responsible for the transport of a total of 16 different substrates. We were interested in the correlation of these transporter substrates with the predicted metabolic network of *P. aeruginosa*. Mapping of these substrates onto the PseudoCyc database [28] indicated that 15 out of the 16 substrates were starting inputs of predicted metabolic pathways in *P. aeruginosa*.

**Figure 4:**
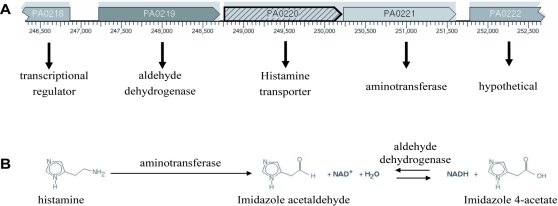
Putative histamine uptake and utilization operon in *P. aeruginosa*. A) Diagrammatic representation of the genetic vicinity of the putative histamine transporter gene PA0220. Each gene is labeled with its predicted function, and the predicted operon structure is indicated with grey shading. B) Illustration of the proposed biochemical pathway for histamine utilization encoded for by this putative operon.

The one exception was histamine. Various soil microorganisms are capable of breaking down histamine, typically via histamine oxidase [29]. However, there are no homologues of this enzyme present in *P. aeruginosa* or any other sequenced pseudomonad. A monoamine oxidase has been reported previously in *P. aeruginosa*, however, histamine was not a substrate for this enzyme, nor was it induced by histamine [30]. The putative histamine transporter gene PA0220 is encoded in a gene cluster with putative aldehyde dehydrogenase and aminotransferase genes of unknown specificity (Figure 4a). Speculatively this gene cluster may represent an operon encoding a transporter for histamine, and a two-step catabolic pathway that would convert histamine to imidazole-4-acetate (Figure 4b). Imidazole-4-acetate might then conceivably be fed into aspartate metabolism via conversion to imidazolone acetate and subsequently to N-formyl-L-aspartate. Supporting this hypothesis, qRT-PCR analysis indicated that expression of both PA0219 and PA0221 are induced 40-fold by histamine. PA0218 is a putative transcriptional regulator gene that is divergently transcribed from this putative histamine utilization operon and might be responsible for its regulation.

## Discussion

The use of Biolog phenotype MicroArray plates PM1-10 allowed us to determine almost 1000 phenotypic traits for 78 individual knockout mutants. These experiments in *P. aeruginosa* PAO1 identified a total of 136 different compounds that could be utilized as carbon sources and 351 different compounds that could be utilized as nitrogen sources (Table S2). Comparisons of substrate utilization capabilities of mutants and isogenic parent strains allowed significant phenotypes to be assigned to 27 of the genes tested. For each mutant the phenotype was subsequently confirmed by independent growth studies on minimal media plates.

Based on these results it is possible to assess the accuracy of the initial bioinformatic predictions. In some cases the experimental data and the bioinformatic predictions essentially correlate. In other cases bioinformatics was able to make only a very general prediction, and experimental data was necessary to clarify the precise substrate specificity of the transporter. Of the 27 successfully characterized genes predicted to encode transporter-related functions, only five annotations were revised based on experimental data. Of these, three genes are part of a single operon (PA1256, PA1257 and PA1260) which was predicted to be involved in amino acid transport and instead found to be involved in Hydroxy-L-Proline transport. The predicted function of PA3176 (gltS) was likewise modified slightly, with bioinformatic predictions suggesting a role as a glutamate/sodium symporter, while experimental data indicated transport of N-acetyl-glutamate. Perhaps the most interesting deviation from the bioinformatic prediction involves PA0220, which, while originally predicted to function in amino acid uptake, based on the Biolog data probably functions in histamine transport. To our knowledge this is the first identification of a histamine transporter gene in a bacterial species.

In the remaining 22 instances, bioinformatic predictions correlated well with the characterization results (12/27) or provided a correct start point, which experimental findings added to, giving a more precise functional assignment (10/27). These findings indicate that bioinformatic predictions are a valuable starting point in determining gene functions. However, such approaches are not infallible and in many cases can provide only a generalized function prediction, highlighting the need for high throughput experimental approaches to confirm and add detail to phenotypic predictions.

The Biolog PM system has been proposed to provide a rapid means of evaluating phenotypic predictions for large sets of genes [8]. To date, however, there has only been one published account of a study using the PM system to undertake a large scale functional screen for a specific subset of genes. Zhou et al (2003) used this system to investigate two-component regulatory systems in *Escherichia coli* K-12, screening mutants in 37 different two-component genes for altered growth on PM1-20 plates [31]. Altered phenotypes were observed for 22 of the 37 different two-component mutants, in most instances these were as expected, however several new phenotypes were revealed. This was a higher rate of mutant phenotype identification than what we observed for *P. aeruginosa* transporter mutants, but this is probably due to the likelihood that regulatory gene mutants have more pleiotropic effects than transporter gene knockouts.

In this study the Biolog PM system allowed phenotypes to be assigned to 27 of the 78 genes tested. While the Biolog assays performed in this study did not assign specific functions to all putative transporter genes, these experiments did provide indirect evidence of the transporter capacity of *P. aeruginosa*. Both studied strains of *P. aeruginosa* were observed to grow on all tested amino acids indicating that specific or generic transporters are likely to exist for each. Our experimental analyses were successful in characterizing transporters for seven of the standard amino acids. A greater degree of success was obtained with characterization of the two clusters of genes predicted to comprise sugar phosphotransferase system transporters, with their roles confirmed and specific substrates identified for each.

There are a number of potential reasons why only a proportion of the *P. aeruginosa* amino acid transporters were able to be experimentally identified. We may not have knockout mutants in all *P. aeruginosa* amino acid transporter genes, possibly because they are essential for cell survival or are novel transporter types that were not identified in our bioinformatic screen. For some substrates there may be multiple transporters responsible for their uptake, in which case loss of one of these transport pathways through a knockout mutation may not reduce uptake sufficiently to be detected with these assays. The well-specific variability in the Biolog assays observed for some compounds, such as cysteine, probably precluded the identification of any transporters for those substrates. Another general issue for this type of approach is that the substrates of particular transporter systems may not be one of the 2000 phenotypes currently tested by Biolog plates.

While there are limitations to what the Biolog PM system can achieve, utilizing this tool allowed many more mutants to be screened (and on vastly more substrates) than would have been possible with any other current approach. This has allowed the characterization of a total of 27 transporter genes in a single study, a considerable undertaking when most characterization studies to date have tackled only one or two such operons at a time. One notable exception is the recent study by Tamber et al (2006), which aimed to characterize 17 genes predicted to encode porins involved in nutrient uptake in *P. aeruginosa* [20]. This study characterized knockout mutants, defective in putative *opr*D family genes, obtained from the same mutant collection we utilized. In this case standard growth experiments, rather than Phenotype MicroArrays, were used to determine the associated phenotypes, providing an interesting parallel to the characterization approach presented here. The authors of this study were able to confirm the predicted functions of six of these 17 *opr*D family genes, a similar proportion to what was achieved in this study while a smaller overall number.

This comparison highlights another advantage of the Biolog system. Standard plate growth assays can be set up easily and cheaply to confirm gene functions where reasonably specific bioinformatic predictions of function are available, limiting the number of growth substrates to be tested. However, this approach is not suitable where there are no bioinformatic “clues” regarding specificity of the putative transporter, an issue overcome with the Biolog system, which allows rapid screening of thousands of potential compounds simultaneously. Such comprehensive screening also makes it possible to detect novel transporters, such as the histamine transporter detected in this study, which would have been unlikely to have been uncovered by more directed growth assays.

In this study, the Biolog PM system was used to screen a significant number of mutants for changes in growth on a very large number of substrates. One of the most significant advantages of this approach was the capacity to rapidly characterize transporters for which bioinformatic predictions provide only a general indication of function, a feat which previously would have required a much greater expenditure of time and research effort.

## Materials and Methods

### Bacterial Strains and Media

All strains employed in this study are listed in supporting Table S3. The 384 *P. aeruginosa* Mini-Tn*5*-Tc^r^ transporter gene knockout mutants were obtained from PathoGenesis Corporation (Seattle, WA). The liquid minimal growth medium was M9 salts plus 20 mM sodium succinate, 2 µM ferric citrate, 1 mM magnesium sulfate, 0.1 mM calcium chloride. Minimal medium plates were made with 1.5% agar.

### Bioinformatic Analysis

Functional predictions of membrane transporter genes were assigned by running the complete *P. aeruginosa* gene set through our automated annotation pipeline [6], which utilizes a series of BLAST, HMM and COG-based searches in conjunction with other analyses such as transmembrane segment prediction, followed by careful manual curation. Additionally, phylogenetic trees for every transporter protein were generated, and a comparative analysis of the genomic context (i.e., the flanking genes) was undertaken, in order to update the annotation of *P. aeruginosa* transporter genes.

### Phenotype MicroArray Assays

Strains to be tested were plated on Biolog Universal Growth medium plus Blood agar plates and incubated overnight at 37°C. Cells were swabbed from the plates after overnight growth and suspended in appropriate medium containing Dye Mix C; 100 µL of a 1∶200 dilution of an 85% transmittance suspension of cells were added to each well of the PM plates. Plates 1–8, which test for catabolic pathways for carbon, nitrogen, phosphorus, sulfur, as well as for biosynthetic pathways, and plates 9–10, which test for osmotic/ion and pH effects, were utilized in this study. IF-0 GN Base was used for PM plates 1 and 2. IF-0 GN Base plus 20 mM sodium succinate, pH 7.1, and 2 µM ferric citrate was used for plates 3–8. IF-10 Base was used for plates 9 and 10. Plates were incubated in the OmniLog for 48 hours with readings taken every 15 minutes. Data analysis was performed using Kinetic and Parametric software (Biolog). Phenotypes were determined based on the area difference under the kinetic curve of dye formation between the mutant and wild type. Data points for the entire 48 hours were used for PM1 through PM8, and area differences were mean-centered by plate. PM4 was subdivided into phosphorus utilization and sulfur utilization sections, and these were mean-centered individually. Only data points from the first 24 hours were used for PM9 and PM10, and area differences were not mean-centered. PAK and PAO1 strains were grouped separately, and mean values were determined for each well. Values beyond two standard deviations from the mean were considered for further analysis.

### Minimal Media Growth Assays

Wild type and mutant strains were grown on minimal medium agar plates containing either the predicted substrate substituted for sodium succinate and ferric citrate when testing carbon sources, or the predicted substrate substituted for ammonium chloride when testing nitrogen sources. Each strain was also grown on minimal media plates without substitutions as a control. Growth phenotypes were determined based on isolated colony sizes, with assays performed in triplicate.

### Quantitative RT-PCR

The ability of putative substrates to induce transporter gene expression in the wild type PAK and PAO1 strains was assessed by quantitative real-time polymerase chain reaction (qRT-PCR). Overnight liquid minimal medium cultures were grown for an additional two hours in the presence of 0.1% (w/v) of the potential inducer, and RNA was extracted using Trizol and was further purified using the RNeasy Mini Kit (Qiagen). RNA underwent DNase digestion using the DNA-free Turbo DNase Digestion Kit (Ambion). qRT-PCR reactions were carried out using the Superscript III Platinum SYBR Green One-Step qRT-PCR Kit (Invitrogen) in an ABI PRISM 7900(HT). Primers were designed with Primer3 [32] for products of 100–250 bp in length. As an internal control, RT-PCR was carried out using primers for the amplification of *rplU* which encodes 50S ribosomal protein L21. Reactions were performed at least once from each of three biological replicates. Cycle threshold (CT) values for each reaction were determined with ABI PRISM SDS 2.1 software. Analysis of relative RNA abundance was performed using the ΔΔC_T_ method (PE Applied Biosystems).

## Supporting Information

Table S1 List of bioinformatic predictions for possible transporters.
(0.75 MB DOC)

Table S2 *Pseudomonas aeruginosa* PA01 substrate utilization profiles from Biolog phenotype MicroArrays.
(1.42 MB DOC)

Table S3 Strain list.
(0.49 MB DOC)
