# Supplementary material for: High-Throughput Phenotypic Characterization of Pseudomonas aeruginosa Membrane Transport Genes
Source: PLoS Genet. 2008 Oct 3;4(10):e1000211. doi: 10.1371/journal.pgen.1000211 (PMC2542419; doi:10.1371/journal.pgen.1000211)
Supplement: Table S1 — List of bioinformatic predictions for possible transporters. (0.75 MB DOC) [file pgen.1000211.s001.doc]

**Table S1.** List of bioinformatic predictions for possible transporters.

| ***ORF*** | ***TC family^a^*** | ***Predicted Substrate*** | ***Original Annotation^b^*** |
| --- | --- | --- | --- |
| PA0029 | SulP | sulfate | probable sulfate transporter |
| PA0030 | ABC | glycine/betaine | **hypothetical protein** |
| PA0072 | ABC |  | **hypothetical protein** |
| PA0073 | ABC |  | probable ATP-binding component of ABC transporter |
| PA0103 | SulP | sulfate | probable sulfate transporter |
| PA0119 | DAACS | C4-dicarboxylate | probable dicarboxylate transporter |
| PA0129 | APC | gamma-aminobutyrate | gamma-aminobutyrate permease |
| PA0136 | ABC | sugar | probable ATP-binding component of ABC transporter |
| PA0137 | ABC | sugar | probable permease of ABC transporter |
| PA0138 | ABC | sugar | probable permease of ABC transporter |
| PA0146 | ABC | sugar | **conserved hypothetical protein** |
| PA0158 | RND | acriflavin efflux | probable RND efflux transporter |
| PA0166 | NCS2 | xanthine/uracil | probable transporter |
| PA0184 | ABC | sulfate ester | probable ATP-binding component of ABC transporter |
| PA0185 | ABC | sulfate ester | probable permease of ABC transporter |
| PA0186 | ABC | sulfate ester | probable binding protein component of ABC transporter |
| PA0188 | AEC |  | **hypothetical protein** |
| PA0203 | ABC | spermidine/putrescine | probable binding protein component of ABC transporter |
| PA0204 | ABC | spermidine/putrescine | probable permease of ABC transporter |
| PA0205 | ABC | spermidine/putrescine | probable permease of ABC transporter |
| PA0206 | ABC | spermidine/putrescine | probable ATP-binding component of ABC transporter |
| PA0215 | MSS | malonate/sodium ion symporter MadL | probable transporter |
| PA0216 | MSS | malonate/sodium ion symporter MadM | probable transporter |
| PA0220 | APC | amino acid | probable amino acid permease |
| PA0222 | ABC | polyamine | **hypothetical protein** |
| PA0229 | MFS | dicarboxylic acid | dicarboxylic acid transporter PcaT |
| PA0235 | MFS | multidrug efflux | 4-hydroxybenzoate transporter PcaK |
| PA0239 | DMT | drug/metabolite | **hypothetical protein** |
| PA0241 | MFS | hexuronate | probable MFS transporter |
| PA0246 | MFS | multidrug efflux | probable MFS transporter |
| PA0273 | MFS | cyanate | probable MFS transporter |
| PA0280 | ABC | sulfate | sulfate transport protein CysA |
| PA0281 | ABC | sulfate | sulfate transport protein CysW |
| PA0282 | ABC | sulfate | sulfate transport protein CysT |
| PA0283 | ABC | sulfate | sulfate-binding protein precursor |
| PA0287 | SSS | sodium ion/solute | probable sodium:solute symporter |
| PA0295 | ABC | putrescine | probable periplasmic polyamine binding protein |
| PA0300 | ABC | putrescine | polyamine transport protein |
| PA0301 | ABC | putrescine | polyamine transport protein |
| PA0302 | ABC | putrescine | polyamine transport protein PotG |
| PA0303 | ABC | putrescine | polyamine transport protein PotH |
| PA0304 | ABC | putrescine | polyamine transport protein PotI |
| PA0313 | ABC | amino acid (cystine) | probable permease of ABC transporter |
| PA0314 | ABC | amino acid (cystine) | probable binding protein component of ABC transporter |
| PA0322 | APC | amino acid | probable transporter |
| PA0323 | ABC | spermidine/putrescine | probable binding protein component of ABC transporter |
| PA0324 | ABC | spermidine/putrescine | probable permease of ABC transporter |
| PA0325 | ABC | spermidine/putrescine | probable permease of ABC transporter |
| PA0326 | ABC | spermidine/putrescine | probable ATP-binding component of ABC transporter |
| PA0334 | MFS | multidrug efflux | probable MFS transporter |
| PA0337 | GPTS |  | phosphoenolpyruvate-protein phosphotransferase PtsP |
| PA0352 | NCS2 | xanthine/uracil | probable transporter |
| PA0373 | IISP | signal recognition particle receptor FtsY | signal recognition particle receptor FtsY |
| PA0374 | ABC | cell division | cell division protein FtsE |
| PA0375 | ABC | cell division | cell division protein FtsX |
| PA0397 | CDF | cobalt/cadmium/zinc ion efflux | probable cation efflux system protein |
| PA0417 | RhtB | amino acid efflux | probable chemotaxis protein |
| PA0426 | RND | multiple drug/N-(3-oxododecanoyl)- L-homoserine lactone autoinducer efflux pump | RND multidrug efflux transporter MexB |
| PA0438 | NCS1 | cytosine | cytosine permease |
| PA0443 | NCS1 | cytosine/purines/uracil/thiamine/allantoin | probable transporter |
| PA0450 | PiT | phosphate | probable phosphate transporter |
| PA0454 | ArAE |  | **conserved hypothetical protein** |
| PA0458 | MFS | multidrug efflux | probable MFS transporter |
| PA0476 | NCS1 | cytosine/purines/uracil/thiamine/allantoin | probable permease |
| PA0485 | DMT | chloramphenicol (RarD) | **conserved hypothetical protein** |
| PA0539 | DMT | drug/metabolite | **hypothetical protein** |
| PA0545 | CytB | oxidoreductase | **hypothetical protein** |
| PA0602 | ABC | polyamine | probable binding protein component of ABC transporter |
| PA0603 | ABC | polyamine | probable ATP-binding component of ABC transporter |
| PA0604 | ABC | polyamine | probable binding protein component of ABC transporter |
| PA0605 | ABC | polyamine | probable permease of ABC transporter |
| PA0606 | ABC | polyamine | probable permease of ABC transporter |
| PA0688 | ABC | phosphate | **hypothetical protein** |
| PA0689 | ABC | phosphate | **hypothetical protein** |
| PA0703 | MFS |  | probable MFS transporter |
| PA0716 | ABC | amino acid | **hypothetical protein** |
| PA0742 | VIC | potassium ion channel | **hypothetical protein** |
| PA0752 | TTT | tricarboxylate (TctA) | **conserved hypothetical protein** |
| PA0753 | TTT | tricarboxylate (TctB) | **hypothetical protein** |
| PA0754 | TTT | tricarboxylate (TctC) | **hypothetical protein** |
| PA0777 | ABC | amino acid | **hypothetical protein** |
| PA0783 | SSS | sodium ion/proline | sodium/proline symporter PutP |
| PA0786 | DMT | quaternary ammonium compound SugE | probable transporter |
| PA0789 | APC | amino acid (proline) | probable amino acid permease |
| PA0809 | Nramp | manganese ion | probable transporter |
| PA0811 | MFS |  | probable MFS transporter |
| PA0860 | ABC | multidrug | probable ATP-binding/permease fusion ABC transporter |
| PA0866 | APC | aromatic amino acid | aromatic amino acid transport protein AroP2 |
| PA0875 | ArAE | fusaric acid efflux | **conserved hypothetical protein** |
| PA0884 | TRAP-T | C4-dicarboxylate | probable C4-dicarboxylate-binding periplasmic protein |
| PA0885 | TRAP-T | C4-dicarboxylate | probable C4-dicarboxylate transporter |
| PA0886 | TRAP-T | C4-dicarboxylate | probable C4-dicarboxylate transporter |
| PA0888 | ABC | arginine/ornithine (but not lysine) porter | arginine/ornithine binding protein AotJ |
| PA0889 | ABC | arginine/ornithine (but not lysine) porter | arginine/ornithine transport protein AotQ |
| PA0890 | ABC | arginine/ornithine (but not lysine) porter | arginine/ornithine transport protein AotM |
| PA0892 | ABC | arginine/ornithine (but not lysine) porter | arginine/ornithine transport protein AotP |
| PA0913 | MgtE | magnesium ion | probable Mg transporter MgtE |
| PA0917 | KUP | potassium ion uptake | potassium uptake protein Kup |
| PA1019 | MFS | cis,cis-muconate | cis,cis-muconate transporter MucK |
| PA1051 | GntP | gluconate | probable transporter |
| PA1054 | CPA3 | sodium ion/proton | probable NADH dehydrogenase |
| PA1055 | CPA3 | sodium ion/proton | **conserved hypothetical protein** |
| PA1056 | CPA3 | sodium ion/proton | probable NADH dehydrogenase |
| PA1057 | CPA3 | sodium ion/proton | **conserved hypothetical protein** |
| PA1058 | CPA3 | sodium ion/proton | **conserved hypothetical protein** |
| PA1059 | CPA3 | sodium ion/proton | **conserved hypothetical protein** |
| PA1060 | DMT | drug/metabolite | **hypothetical protein** |
| PA1070 | ABC | branched-chain amino acid | branched-chain amino acid transport protein BraG |
| PA1071 | ABC | branched-chain amino acid | branched-chain amino acid transport protein BraF |
| PA1072 | ABC | branched-chain amino acid | branched-chain amino acid transport protein BraE |
| PA1073 | ABC | branched-chain amino acid | branched-chain amino acid transport protein BraD |
| PA1074 | ABC | branched-chain amino acid | branched-chain amino acid transport protein BraC |
| PA1108 | MFS | multidrug efflux | probable MFS transporter |
| PA1113 | ABC | multidrug | probable ATP-binding/permease fusion ABC transporter |
| PA1131 | MFS | tetracycline efflux | probable MFS transporter |
| PA1144 | MFS | sugar | probable MFS transporter |
| PA1147 | APC | amino acid | probable amino acid permease |
| PA1183 | DAACS | C4-dicarboxylate | C4-dicarboxylate transport protein |
| PA1194 | APC | arginine/ornithine antiporter | probable amino acid permease |
| PA1207 | CPA2 | glutathione-regulated potassium ion efflux | glutathione-regulated potassium-efflux system protein KefB |
| PA1212 | MFS |  | probable MFS transporter |
| PA1232 | ArAE | fusaric acid efflux | **hypothetical protein** |
| PA1236 | MFS | multidrug efflux | probable MFS transporter |
| PA1246 | ABC | alkaline protease exporter | alkaline protease secretion protein AprD |
| PA1256 | ABC | amino acid (glutamine) | probable ATP-binding component of ABC transporter |
| PA1257 | ABC | amino acid (glutamine) | probable permease of ABC transporter |
| PA1258 | ABC | amino acid (glutamine) | probable permease of ABC transporter |
| PA1260 | ABC | amino acid (glutamine) | probable binding protein component of ABC transporter |
| PA1262 | MFS |  | probable MFS transporter |
| PA1265 | DMT | drug/metabolite | **hypothetical protein** |
| PA1270 | ArAE | fusaric acid efflux | **hypothetical protein** |
| PA1282 | MFS | multidrug efflux (methyl viologen resistance) | probable MFS transporter |
| PA1286 | MFS | oxalate/formate | probable MFS transporter |
| PA1297 | CDF | heavy metal ion efflux | probable metal transporter |
| PA1313 | MFS | multidrug efflux | probable MFS transporter |
| PA1316 | MFS | multidrug efflux | probable MFS transporter |
| PA1339 | ABC | amino acid (glutamate/aspartate) | probable ATP-binding component of ABC transporter |
| PA1340 | ABC | amino acid (glutamate/aspartate) | probable permease of ABC transporter |
| PA1341 | ABC | amino acid (glutamate/aspartate) | probable permease of ABC transporter |
| PA1342 | ABC | amino acid (glutamate/aspartate) | probable binding protein component of ABC transporter |
| PA1352 | MFS |  | **conserved hypothetical protein** |
| PA1360 | DMT | drug/metabolite | **conserved hypothetical protein** |
| PA1361 | MOP | drug:H+ antiporter (benzalkonium chloride, fluoroquinolone, ethidium bromide, acriflavin, tetrapheny | probable transporter |
| PA1386 | ABC | O-antigen/polysaccharide | probable ATP-binding component of ABC transporter |
| PA1408 | MscS | small-conductance mechanosensitive ion channel | **hypothetical protein** |
| PA1410 | ABC | putrescine | probable periplasmic spermidine/putrescine-binding protein |
| PA1411 | DMT | drug/metabolite | **hypothetical protein** |
| PA1412 | MFS |  | **hypothetical protein** |
| PA1418 | SSS | sodium ion/solute | probable sodium:solute symport protein |
| PA1419 | NCS1 | cytosine/purines/uracil/thiamine/allantoin | probable transporter |
| PA1425 | ABC | Uup homolog/duplicated ATPase | probable ATP-binding component of ABC transporter |
| PA1429 | P-ATPase | cation | probable cation-transporting P-type ATPase |
| PA1436 | RND | acriflavin efflux | probable RND efflux transporter |
| PA1453 | IISP | flagellar biosynthesis protein FlhF | flagellar biosynthesis protein FlhF |
| PA1475 | ABC | heme | heme exporter protein CcmA |
| PA1476 | ABC | heme | heme exporter protein CcmB |
| PA1477 | ABC | heme | heme exporter protein CcmC |
| PA1485 | APC | amino acid | probable amino acid permease |
| PA1493 | ABC | sulfate/thiosulphate | sulfate-binding protein of ABC transporter |
| PA1496 | VIC | potassium ion channel | probable potassium channel |
| PA1497 | UT | urea | probable transporter |
| PA1507 | NCS2 | xanthine/uracil | probable transporter |
| PA1519 | NCS2 | xanthine/uracil | probable transporter |
| PA1531 | ABC | amino acid | **hypothetical protein** |
| PA1540 | DMT | multidrug | **conserved hypothetical protein** |
| PA1541 | DMT | multidrug | probable drug efflux transporter |
| PA1549 | P-ATPase | cation | probable cation-transporting P-type ATPase |
| PA1569 | MFS | glycerol-3-phosphate | probable MFS transporter |
| PA1590 | LIVCS | branched-chain amino acid:sodium symporter | branched chain amino acid transporter |
| PA1612 | ABC |  | **hypothetical protein** |
| PA1620 | RhtB | threonine efflux | **hypothetical protein** |
| PA1626 | MFS | cyanate | probable MFS transporter |
| PA1633 | P-ATPase | potassium ion | potassium-transporting ATPase, A chain |
| PA1634 | P-ATPase | potassium ion | potassium-transporting ATPase, B chain |
| PA1635 | P-ATPase | potassium ion | potassium-transporting ATPase, C chain |
| PA1647 | SulP | sulfate | probable sulfate transporter |
| PA1650 | BASS | sodium ion/bile acid | probable transporter |
| PA1651 | BenE | benzoate | probable transporter |
| PA1682 | MFS | nucleoside:proton symporter | probable MFS metabolite transporter |
| PA1735 | AEC |  | **hypothetical protein** |
| PA1773 | MIT | magnesium/cobalt ion | **conserved hypothetical protein** |
| PA1775 | MscS | small-conductance mechanosensitive ion channel | **conserved hypothetical protein** |
| PA1783 | MFS | nitrate | nitrate transporter |
| PA1786 | ABC | nitrate | **conserved hypothetical protein** |
| PA1807 | ABC | Uup homolog/duplicated ATPase | probable ATP-binding component of ABC transporter |
| PA1808 | ABC | peptide | probable permease of ABC transporter |
| PA1809 | ABC | peptide | probable permease of ABC transporter |
| PA1810 | ABC | peptide | probable binding protein component of ABC transporter |
| PA1811 | ABC | peptide | probable solute-binding protein |
| PA1819 | APC | amino acid | probable amino acid permease |
| PA1820 | NhaB | sodium ion/proton | sodium/proton antiporter NhaB |
| PA1848 | MFS | multidrug/metabolite efflux | probable MFS transporter |
| PA1861 | ABC | molybdenum | molybdenum transport protein ModC |
| PA1862 | ABC | molybdenum | molybdenum transport protein ModB |
| PA1863 | ABC | molybdenum | molybdate-binding periplasmic protein precursor ModA |
| PA1873 | MIT | magnesium/cobalt ion | **hypothetical protein** |
| PA1876 | ABC | toxin secretion | probable ATP-binding/permease fusion ABC transporter |
| PA1882 | DMT | quaternary ammonium compound SugE | probable transporter |
| PA1908 | MFS | multidrug efflux | probable MFS transporter |
| PA1916 | HAAAP | aromatic amino acid | probable amino acid permease |
| PA1946 | ABC | ribose | binding protein component precursor of ABC ribose transporter |
| PA1947 | ABC | ribose | ribose transport protein RbsA |
| PA1948 | ABC | ribose | membrane protein component of ABC ribose transporter |
| PA1958 | PnuC | nicotinamide mononucleotide | probable transporter |
| PA1964 | ABC | Uup homolog/duplicated ATPase | probable ATP-binding component of ABC transporter |
| PA1971 | LIVCS | Ileucine/Valine:proton symporter | branched chain amino acid transporter BraZ |
| PA1977 | DMT | drug/metabolite | **hypothetical protein** |
| PA1993 | MFS |  | probable MFS transporter |
| PA2002 | MFS | short-chain fatty acids | **conserved hypothetical protein** |
| PA2004 | GntP | gluconate | **conserved hypothetical protein** |
| PA2006 | MFS | 4-hydroxybenzoate | probable MFS transporter |
| PA2018 | RND | multidrug (aminoglycosides, tetracycline, erythromycin, ofloxacin, etc.) efflux pump (MexY) | RND multidrug efflux transporter |
| PA2026 | BASS | sodium ion/bile acid | **conserved hypothetical protein** |
| PA2039 | LIV-E | branched-chain amino acid efflux (AzlC) | **hypothetical protein** |
| PA2041 | APC | amino acid | probable amino acid permease |
| PA2042 | DAACS | sodium ion:dicarboxylate symporter | probable transporter (membrane subunit) |
| PA2055 | MFS | multidrug efflux | probable MFS transporter |
| PA2058 | ABC | peptide | probable binding protein component of ABC transporter |
| PA2059 | ABC | peptide | probable permease of ABC transporter |
| PA2060 | ABC | peptide | probable permease of ABC transporter |
| PA2061 | ABC | peptide | probable ATP-binding component of ABC transporter |
| PA2068 | MFS | 3-phenylpropionic acid | probable MFS transporter |
| PA2073 | NCS1 | cytosine/purines/uracil/thiamine/allantoin | probable transporter (membrane subunit) |
| PA2079 | APC | amino acid | probable amino acid permease |
| PA2091 | MFS |  | **hypothetical protein** |
| PA2092 | MFS | chloramphenicol efflux | probable MFS transporter |
| PA2101 | DMT | drug/metabolite | **conserved hypothetical protein** |
| PA2114 | MFS |  | probable MFS transporter |
| PA2135 | CPA1 | sodium ion/proton | probable transporter |
| PA2202 | ABC | amino acid (glutamine) | probable amino acid permease |
| PA2203 | ABC | amino acid (glutamine) | probable amino acid permease |
| PA2204 | ABC | amino acid (glutamine) | probable binding protein component of ABC transporter |
| PA2207 | TTT | tricarboxylate (TctA) | **hypothetical protein** |
| PA2208 | TTT | tricarboxylate (TctB) | **hypothetical protein** |
| PA2209 | TTT | tricarboxylate (TctC) | **hypothetical protein** |
| PA2210 | MFS | D-galactonate | probable MFS transporter |
| PA2214 | MFS |  | probable MFS transporter |
| PA2219 | MFS | sugar (arabinose) efflux | membrane protein OpdE |
| PA2241 | MOP | virulence factor MviN | **hypothetical protein** |
| PA2252 | AGCS | sodium ion/alanine | probable AGCS sodium/alanine/glycine symporter |
| PA2262 | MFS | 2-ketogluconate | probable 2-ketogluconate transporter |
| PA2269 | MFS |  | **conserved hypothetical protein** |
| PA2278 | ArsB | arsenite (ArsB) | ArsB protein |
| PA2294 | ABC | nitrate/sulfonate/taurine | probable ATP-binding component of ABC transporter |
| PA2295 | ABC | nitrate/sulfonate/taurine | probable permease of ABC transporter |
| PA2296 | ABC | nitrate/sulfonate/taurine | **hypothetical protein** |
| PA2306 | RhtB | amino acid efflux | **conserved hypothetical protein** |
| PA2307 | ABC | nitrate/sulfonate/taurine | probable permease of ABC transporter |
| PA2308 | ABC | nitrate/sulfonate/taurine | probable ATP-binding component of ABC transporter |
| PA2309 | ABC | nitrate/sulfonate/taurine | **hypothetical protein** |
| PA2314 | MFS |  | probable MFS transporter |
| PA2322 | GntP | gluconate | gluconate permease |
| PA2327 | ABC | nitrate/sulfonate/taurine | probable permease of ABC transporter |
| PA2328 | ABC | nitrate/sulfonate/taurine | **hypothetical protein** |
| PA2329 | ABC | nitrate/sulfonate/taurine | probable ATP-binding component of ABC transporter |
| PA2338 | ABC | maltose/mannitol | probable binding protein component of ABC maltose/mannitol transporter |
| PA2339 | ABC | maltose/mannitol | probable binding-protein-dependent maltose/mannitol transport protein |
| PA2340 | ABC | maltose/mannitol | probable binding-protein-dependent maltose/mannitol transport protein |
| PA2341 | ABC | maltose/mannitol | probable ATP-binding component of ABC maltose/mannitol transporter |
| PA2349 | ABC | D-methionine | **conserved hypothetical protein** |
| PA2350 | ABC | D-methionine | probable ATP-binding component of ABC transporter |
| PA2351 | ABC | D-methionine | probable permease of ABC transporter |
| PA2377 | ABC |  | **hypothetical protein** |
| PA2390 | ABC | macrolide efflux | probable ATP-binding/permease fusion ABC transporter |
| PA2397 | ABC | pyoverdin (siderophore) exporter PvdE | pyoverdine biosynthesis protein PvdE |
| PA2407 | ABC | manganese/zinc ion | probable adhesion protein |
| PA2408 | ABC | manganese/zinc ion | probable ATP-binding component of ABC transporter |
| PA2409 | ABC | manganese/zinc ion | probable permease of ABC transporter |
| PA2410 | ABC | manganese/zinc ion | **hypothetical protein** |
| PA2431 | ArAE | fusaric acid efflux | **hypothetical protein** |
| PA2435 | P-ATPase | cadmium ion | probable cation-transporting P-type ATPase |
| PA2472 | MFS | 4-hydroxybenzoate | probable MFS transporter |
| PA2494 | RND | multidrug (xenobiotics/chloramphenicol)efflux (MexF) | RND multidrug efflux transporter MexF |
| PA2500 | MFS | cyanate | probable MFS transporter |
| PA2520 | RND | cobalt/zinc/cadmium ion efflux | RND divalent metal cation efflux transporter CzcA |
| PA2526 | RND | multidrug efflux | probable RND efflux transporter |
| PA2527 | RND | multidrug efflux | probable RND efflux transporter |
| PA2533 | AGCS | sodium ion/alanine | probable sodium:alanine symporter |
| PA2563 | SulP | sulfate | probable sulfate transporter |
| PA2576 | DMT | drug/metabolite | **hypothetical protein** |
| PA2589 | MFS |  | **hypothetical protein** |
| PA2592 | ABC | polyamine | probable periplasmic spermidine/putrescine-binding protein |
| PA2594 | ABC | sulfonate | **conserved hypothetical protein** |
| PA2595 | ABC | sulfonate | **conserved hypothetical protein** |
| PA2596 | ABC | sulfonate | **conserved hypothetical protein** |
| PA2599 | ABC | sulfonate | **conserved hypothetical protein** |
| PA2628 | DMT | drug/metabolite | **hypothetical protein** |
| PA2653 | NhaC | sodium ion/proton | probable transporter |
| PA2678 | ABC | O-antigen | probable permease of ABC-2 transporter |
| PA2701 | MFS |  | probable MFS transporter |
| PA2710 | RhtB | amino acid efflux | **hypothetical protein** |
| PA2711 | ABC | putrescine | probable periplasmic spermidine/putrescine-binding protein |
| PA2712 | DMT | drug/metabolite | **hypothetical protein** |
| PA2777 | FNT | formate/nitrite | **conserved hypothetical protein** |
| PA2811 | ABC | multidrug | probable permease of ABC-2 transporter |
| PA2812 | ABC | multidrug | probable ATP-binding component of ABC transporter |
| PA2835 | MFS | multidrug efflux | probable MFS transporter |
| PA2857 | ABC |  | probable ATP-binding component of ABC transporter |
| PA2858 | ABC |  | **conserved hypothetical protein** |
| PA2902 | ABC | amino acid | **hypothetical protein** |
| PA2912 | ABC | iron compound | probable ATP-binding component of ABC transporter |
| PA2913 | ABC | iron compound | **hypothetical protein** |
| PA2914 | ABC | iron compound | probable permease of ABC transporter |
| PA2916 | RhtB | amino acid efflux | **hypothetical protein** |
| PA2923 | ABC | histidine | periplasmic histidine-binding protein HisJ |
| PA2924 | ABC | histidine | histidine transport system permease HisQ |
| PA2925 | ABC | histidine | histidine transport system permease HisM |
| PA2926 | ABC | histidine | histidine transport protein HisP |
| PA2929 | RhtB | amino acid efflux | **hypothetical protein** |
| PA2933 | MFS | sugar efflux | probable MFS transporter |
| PA2938 | NCS2 | xanthine/uracil | probable transporter |
| PA2986 | ABC | lipoprotein releasing | **conserved hypothetical protein** |
| PA2987 | ABC | lipoprotein releasing | probable ATP-binding component of ABC transporter |
| PA2988 | ABC | lipoprotein releasing | **conserved hypothetical protein** |
| PA3000 | APC | aromatic amino acid | aromatic amino acid transport protein AroP1 |
| PA3019 | ABC | Uup homolog/duplicated ATPase | probable ATP-binding component of ABC transporter |
| PA3039 | Amt | ammonium | probable transporter |
| PA3079 | RND |  | **hypothetical protein** |
| PA3137 | MFS | multidrug efflux | probable MFS transporter |
| PA3153 | MOP | O-antigen | O-antigen translocase |
| PA3176 | ESS | sodium ion/glutamate | sodium/glutamate symporter GltS |
| PA3187 | ABC | sugar | probable ATP-binding component of ABC transporter |
| PA3188 | ABC | sugar | probable permease of ABC sugar transporter |
| PA3189 | ABC | sugar | probable permease of ABC sugar transporter |
| PA3190 | ABC | sugar | probable binding protein component of ABC sugar transporter |
| PA3210 | Trk | potassium ion uptake | potassium uptake protein TrkH |
| PA3211 | ABC |  | probable permease of ABC transporter |
| PA3212 | ABC |  | probable ATP-binding component of ABC transporter |
| PA3213 | ABC | toluene tolerance | **hypothetical protein** |
| PA3222 | DMT | drug/metabolite | **hypothetical protein** |
| PA3228 | ABC | multidrug | probable ATP-binding/permease fusion ABC transporter |
| PA3234 | SSS | sodium ion:acetate/glyoxalate | probable sodium:solute symporter |
| PA3236 | ABC | glycine betaine | probable glycine betaine-binding protein precursor |
| PA3250 | ABC | iron(III)/spermidine/putrescine | **hypothetical protein** |
| PA3252 | ABC | iron(III)/spermidine/putrescine | probable permease of ABC transporter |
| PA3253 | ABC | iron(III)/spermidine/putrescine | probable permease of ABC transporter |
| PA3254 | ABC | iron(III)/spermidine/putrescine | probable ATP-binding component of ABC transporter |
| PA3261 | ABC | amino acid | **hypothetical protein** |
| PA3264 | BASS | sodium ion/bile acid | probable transporter |
| PA3265 | DMT | quaternary ammonium compound SugE | probable transporter |
| PA3271 | SSS | nitrogen sensor-receptor domain of the CbrA sensor kinase | probable two-component sensor |
| PA3303 | MFS | chloramphenicol efflux | probable MFS transporter |
| PA3305 | ArAE | fusaric acid efflux | **hypothetical protein** |
| PA3313 | ABC | phosphonate | **hypothetical protein** |
| PA3314 | ABC | phosphonate | probable ATP-binding component of ABC transporter |
| PA3315 | ABC | phosphonate | probable permease of ABC transporter |
| PA3316 | ABC | phosphonate | probable permease of ABC transporter |
| PA3336 | MFS | chloramphenicol efflux | probable MFS transporter |
| PA3355 | MFS |  | **hypothetical protein** |
| PA3358 | DMT | drug/metabolite | **hypothetical protein** |
| PA3362 | UAC | amide | **hypothetical protein** |
| PA3364 | ABC | branched-chain amino acid | aliphatic amidase expression-regulating protein |
| PA3375 | ABC | phosphonate | probable ATP-binding component of ABC transporter |
| PA3376 | ABC | phosphonate | probable ATP-binding component of ABC transporter |
| PA3382 | ABC | phosphonate | phosphonate transport protein PhnE |
| PA3383 | ABC | phosphonate | binding protein component of ABC phosphonate transporter |
| PA3384 | ABC | phosphonate | ATP-binding component of ABC phosphonate transporter |
| PA3393 | ABC | copper ion | NosD protein |
| PA3394 | ABC | copper ion | NosF protein |
| PA3395 | ABC | copper ion | NosY protein |
| PA3400 | ABC | multidrug | **hypothetical protein** |
| PA3401 | ABC | multidrug | **hypothetical protein** |
| PA3406 | ABC | HasA lipase/alkaline protease exporter HasD | transport protein HasD |
| PA3442 | ABC | aliphatic sulfonates | probable ATP-binding component of ABC transporter |
| PA3443 | ABC | aliphatic sulfonates | probable permease of ABC transporter |
| PA3445 | ABC | aliphatic sulfonates | **conserved hypothetical protein** |
| PA3447 | ABC | aliphatic sulfonates | probable ATP-binding component of ABC transporter |
| PA3448 | ABC | aliphatic sulfonates | probable permease of ABC transporter |
| PA3449 | ABC | aliphatic sulfonates | **conserved hypothetical protein** |
| PA3465 | MFS |  | **conserved hypothetical protein** |
| PA3467 | MFS | sugar efflux | probable MFS transporter |
| PA3468 | MscS | small-conductance mechanosensitive ion channel | **conserved hypothetical protein** |
| PA3473 | DMT | chloramphenicol (RarD) | **hypothetical protein** |
| PA3474 | DMT | drug/metabolite | **conserved hypothetical protein** |
| PA3512 | ABC | nitrate/sulfonate/taurine | probable permease of ABC transporter |
| PA3513 | ABC | nitrate/sulfonate/taurine | **hypothetical protein** |
| PA3514 | ABC | nitrate/sulfonate/taurine | probable ATP-binding component of ABC transporter |
| PA3522 | RND | multidrug efflux (MexF) | probable RND efflux transporter |
| PA3532 | MFS |  | **hypothetical protein** |
| PA3538 | ABC | iron(III)/spermidine/putrescine | probable ATP-binding component of ABC transporter |
| PA3557 | DMT | drug/metabolite | **conserved hypothetical protein** |
| PA3558 | DMT | drug/metabolite | **hypothetical protein** |
| PA3560 | SSPTS | fructose | phosphotransferase system, fructose-specific IIBC component |
| PA3562 | SSPTS | fructose | probable phosphotransferase system enzyme I |
| PA3573 | MFS | multidrug efflux | probable MFS transporter |
| PA3581 | MIP | glycerol uptake | glycerol uptake facilitator protein |
| PA3595 | MFS |  | probable MFS transporter |
| PA3597 | APC | amino acid | probable amino acid permease |
| PA3605 | DMT | drug/metabolite | **hypothetical protein** |
| PA3607 | ABC | spermidine/putrescine | polyamine transport protein PotA |
| PA3608 | ABC | spermidine/putrescine | polyamine transport protein PotB |
| PA3609 | ABC | spermidine/putrescine | polyamine transport protein PotC |
| PA3610 | ABC | spermidine/putrescine | polyamine transport protein PotD |
| PA3641 | AGCS | sodium ion/alanine | probable amino acid permease |
| PA3660 | CPA1 | sodium ion/proton | probable sodium/hydrogen antiporter |
| PA3665 | RhtB | amino acid efflux | **hypothetical protein** |
| PA3671 | ABC | multidrug | probable permease of ABC transporter |
| PA3672 | ABC | multidrug | probable ATP-binding component of ABC transporter |
| PA3676 | RND | acriflavin efflux | probable RND efflux transporter |
| PA3690 | P-ATPase | cadmium ion | probable metal-transporting P-type ATPase |
| PA3709 | MFS | sugar efflux | probable MFS transporter |
| PA3718 | MFS |  | probable MFS transporter |
| PA3739 | CPA1 | sodium ion/proton | probable sodium/hydrogen antiporter |
| PA3746 | IISP | signal recognition particle protein Ffh | signal recognition particle protein Ffh |
| PA3749 | MFS | metabolite:proton symporter | probable MFS transporter |
| PA3760 | SSPTS | fructose | probable phosphotransferase protein |
| PA3761 | SSPTS | N-acetylglucosamine | probable phosphotransferase system protein |
| PA3766 | HAAAP | aromatic amino acid | probable aromatic amino acid transporter |
| PA3773 | MFS |  | **hypothetical protein** |
| PA3779 | TRAP-T | C4-dicarboxylate | **hypothetical protein** |
| PA3780 | TRAP-T | C4-dicarboxylate | **hypothetical protein** |
| PA3781 | TRAP-T | C4-dicarboxylate | probable transporter |
| PA3820 | RND | protein-export (SecDF) | secretion protein SecF |
| PA3821 | RND | protein-export (SecDF) | secretion protein SecD |
| PA3836 | ABC |  | **hypothetical protein** |
| PA3837 | ABC |  | probable permease of ABC transporter |
| PA3838 | ABC |  | probable ATP-binding component of ABC transporter |
| PA3839 | DASS | sodium ion/sulfate | probable sodium:sulfate symporter |
| PA3848 | TRAP-T | C4-dicarboxylate | **hypothetical protein** |
| PA3858 | ABC | amino acid | probable amino acid-binding protein |
| PA3865 | ABC | arginine/ornithine | probable amino acid binding protein |
| PA3876 | MFS | nitrite extrusion | nitrite extrusion protein 2 |
| PA3877 | MFS | nitrate extrusion | nitrite extrusion protein 1 |
| PA3887 | CPA1 | sodium ion/proton antiporter NhaP | Na+/H+ antiporter NhaP |
| PA3888 | ABC | glycine betaine/L-proline/carnitine/choline | probable permease of ABC transporter |
| PA3889 | ABC | glycine betaine/L-proline/carnitine/choline | probable binding protein component of ABC transporter |
| PA3890 | ABC | glycine betaine/L-proline/carnitine/choline | probable permease of ABC transporter |
| PA3891 | ABC | glycine betaine/L-proline/carnitine/choline | probable ATP-binding component of ABC transporter |
| PA3893 | ArAE | fusaric acid efflux | **conserved hypothetical protein** |
| PA3897 | DMT | drug/metabolite | **hypothetical protein** |
| PA3920 | P-ATPase | copper ion | probable metal transporting P-type ATPase |
| PA3926 | MFS | multidrug efflux | probable MFS transporter |
| PA3931 | ABC | D-methionine | **conserved hypothetical protein** |
| PA3933 | BCCT | choline | probable choline transporter |
| PA3934 | OPT | oligopeptide | **conserved hypothetical protein** |
| PA3936 | ABC | taurine | probable permease of ABC taurine transporter |
| PA3937 | ABC | taurine | probable ATP-binding component of ABC taurine transporter |
| PA3938 | ABC | taurine | probable periplasmic taurine-binding protein precursor |
| PA3963 | CDF | cation efflux | probable transporter |
| PA4023 | APC | ethanolamine | probable transport protein |
| PA4027 | ABC | amino acid | **hypothetical protein** |
| PA4034 | MIP | water channel | aquaporin Z |
| PA4037 | ABC | multidrug | probable ATP-binding component of ABC transporter |
| PA4038 | ABC | multidrug | **hypothetical protein** |
| PA4045 | ABC | vitamin B12 | **conserved hypothetical protein** |
| PA4049 | ABC | amino acid | **hypothetical protein** |
| PA4064 | ABC |  | probable ATP-binding component of ABC transporter |
| PA4065 | ABC |  | **hypothetical protein** |
| PA4072 | APC | amino acid | probable amino acid permease |
| PA4096 | MFS | 2'4-diacetylphloroglucinol | probable MFS transporter |
| PA4113 | MFS | sugar efflux | probable MFS transporter |
| PA4126 | MFS |  | probable MFS transporter |
| PA4136 | MFS | multidrug efflux | probable MFS transporter |
| PA4143 | ABC | colicin V secretion | probable toxin transporter |
| PA4158 | ABC | ferric enterobactin | ferric enterobactin transport protein FepC |
| PA4159 | ABC | ferric enterobactin | ferrienterobactin-binding periplasmic protein precursor FepB |
| PA4160 | ABC | ferric enterobactin | ferric enterobactin transport protein FepD |
| PA4161 | ABC | ferric enterobactin | ferric enterobactin transport protein FepG |
| PA4187 | MFS | metabolite | probable MFS transporter |
| PA4192 | ABC | amino acid (glutamine) | probable ATP-binding component of ABC transporter |
| PA4193 | ABC | amino acid (glutamine) | probable permease of ABC transporter |
| PA4194 | ABC | amino acid (glutamine) | probable permease of ABC transporter |
| PA4195 | ABC | amino acid (glutamine) | probable binding protein component of ABC transporter |
| PA4207 | RND | multidrug efflux | probable RND efflux transporter |
| PA4218 | MFS | AmpG-related permease | probable transporter |
| PA4222 | ABC |  | probable ATP-binding component of ABC transporter |
| PA4223 | ABC |  | probable ATP-binding component of ABC transporter |
| PA4233 | MFS | multidrug efflux | probable MFS transporter |
| PA4243 | IISP | preprotein translocase SecY subunit | secretion protein SecY |
| PA4287 | DMT | drug/metabolite | **hypothetical protein** |
| PA4289 | CHR | chromate ion | probable transporter |
| PA4292 | PiT | phosphate | probable phosphate transporter |
| PA4334 | Nramp | manganese ion | probable transport protein |
| PA4343 | MFS | proline/betaine | probable MFS transporter |
| PA4355 | MFS |  | probable MFS transporter |
| PA4358 | FeoB | ferrous ion | probable ferrous iron transport protein |
| PA4365 | LysE | lysine/arginine efflux | probable transporter |
| PA4375 | RND | multidrug efflux | probable RND efflux transporter |
| PA4393 | MFS | AmpG-related permease | probable permease |
| PA4394 | MscS | small-conductance mechanosensitive ion channel | **conserved hypothetical protein** |
| PA4453 | ABC | toluene tolerance | **conserved hypothetical protein** |
| PA4454 | ABC | toluene tolerance | **conserved hypothetical protein** |
| PA4455 | ABC | toluene tolerance | probable permease of ABC transporter |
| PA4456 | ABC | toluene tolerance | probable ATP-binding component of ABC transporter |
| PA4460 | ABC | YhbG-YhbN | **conserved hypothetical protein** |
| PA4461 | ABC | YhbG-YhbN | probable ATP-binding component of ABC transporter |
| PA4464 | SSPTS | nitrogen regulatory | nitrogen regulatory IIA protein |
| PA4466 | GPTS |  | probable phosphoryl carrier protein |
| PA4467 | ZIP | zinc ion | **hypothetical protein** |
| PA4496 | ABC | dipeptide | probable binding protein component of ABC transporter |
| PA4497 | ABC | dipeptide | probable binding protein component of ABC transporter |
| PA4500 | ABC | dipeptide | probable binding protein component of ABC transporter |
| PA4502 | ABC | dipeptide | probable binding protein component of ABC transporter |
| PA4503 | ABC | dipeptide | probable permease of ABC transporter |
| PA4504 | ABC | dipeptide | probable permease of ABC transporter |
| PA4505 | ABC | dipeptide | probable ATP-binding component of ABC transporter |
| PA4506 | ABC | dipeptide | probable ATP-binding component of ABC dipeptide transporter |
| PA4507 | RhtB | amino acid efflux | **hypothetical protein** |
| PA4562 | MOP | virulence factor MviN | **conserved hypothetical protein** |
| PA4593 | ABC | lipoprotein-releasing | probable permease of ABC transporter |
| PA4594 | ABC | lipoprotein-releasing | probable ATP-binding component of ABC transporter |
| PA4595 | ABC | Uup homolog/duplicated ATPase | probable ATP-binding component of ABC transporter |
| PA4598 | RND | multidrug efflux pump MexD (exports levofloxacin, carbenicillin, aztreonam, ceftazidime, cefepime, c | RND multidrug efflux transporter MexD |
| PA4614 | MscL | large-conductance mechanosensitive ion channel | conductance mechanosensitive channel |
| PA4616 | TRAP-T | C4-dicarboxylate | probable c4-dicarboxylate-binding protein |
| PA4622 | MFS |  | probable MFS transporter |
| PA4628 | APC | lysine | lysine-specific permease |
| PA4647 | NCS2 | uracil | uracil permease |
| PA4654 | MFS |  | probable MFS transporter |
| PA4687 | ABC | iron(III) | ferric iron-binding periplasmic protein HitA |
| PA4688 | ABC | iron(III) | iron (III)-transport system permease HitB |
| PA4691 | CytB | oxidoreductase homolog | **hypothetical protein** |
| PA4706 | ABC | hemin | probable ATP-binding component of ABC transporter |
| PA4707 | ABC | hemin | probable permease of ABC transporter |
| PA4708 | ABC | hemin | **hypothetical protein** |
| PA4718 | RND | protein-export (SecD) | **hypothetical protein** |
| PA4719 | NCS2 | xanthine/uracil | probable transporter |
| PA4725 | SSS | nitrogen sensor-receptor domain of the CbrA sensor kinase | probable two-component sensor |
| PA4757 | RhtB | amino acid efflux | **conserved hypothetical protein** |
| PA4770 | LctP | L-lactate | L-lactate permease |
| PA4779 | DMT | drug/metabolite | **hypothetical protein** |
| PA4783 | DMT | drug/metabolite | **conserved hypothetical protein** |
| PA4804 | APC | amino acid | probable amino acid permease |
| PA4821 | MOP | multidrug efflux (DNA-damage-inducible protein F) | probable transporter |
| PA4822 | PNaS | sodium ion/phosphate | **hypothetical protein** |
| PA4825 | P-ATPase | magnesium ion | Mg(2+) transport ATPase, P-type 2 |
| PA4834 | DMT | drug/metabolite | **hypothetical protein** |
| PA4858 | ABC | branched-chain amino acid | **conserved hypothetical protein** |
| PA4859 | ABC | branched-chain amino acid | probable permease of ABC transporter |
| PA4860 | ABC | branched-chain amino acid | probable permease of ABC transporter |
| PA4861 | ABC | branched-chain amino acid | probable ATP-binding component of ABC transporter |
| PA4862 | ABC | branched-chain amino acid | probable ATP-binding component of ABC transporter |
| PA4887 | MFS |  | probable MFS transporter |
| PA4900 | MFS | benzoate | probable MFS transporter |
| PA4903 | MFS | vanillate | probable MFS transporter |
| PA4909 | ABC | branched-chain amino acid | probable ATP-binding component of ABC transporter |
| PA4910 | ABC | branched-chain amino acid | probable ATP-binding component of ABC transporter |
| PA4911 | ABC | branched-chain amino acid | probable permease of ABC branched-chain amino acid transporter |
| PA4912 | ABC | branched-chain amino acid | probable permease of ABC branched chain amino acid transporter |
| PA4913 | ABC | branched-chain amino acid | probable binding protein component of ABC transporter |
| PA4925 | MscS | small-conductance mechanosensitive ion channel | **conserved hypothetical protein** |
| PA4981 | APC | amino acid | probable amino acid permease |
| PA4985 | ABC | spermidine/putrescine | **hypothetical protein** |
| PA4990 | DMT | multidrug efflux | SMR multidrug efflux transporter |
| PA4997 | ABC | lipid A export | transport protein MsbA |
| PA5021 | CPA1 | sodium ion/proton (cell volume regulation protein A homolog) | probable sodium/hydrogen antiporter |
| PA5022 | MscS | potassium ion efflux KefA | **conserved hypothetical protein** |
| PA5030 | MFS |  | probable MFS transporter |
| PA5068 | Tat | protein export | translocation protein TatA |
| PA5069 | Tat | protein export | translocation protein TatB |
| PA5070 | Tat | protein export | transport protein TatC |
| PA5074 | ABC | amino acid (glutamine) | probable ATP-binding component of ABC transporter |
| PA5075 | ABC | amino acid (glutamine) | probable permease of ABC transporter |
| PA5076 | ABC | amino acid (glutamine) | probable binding protein component of ABC transporter |
| PA5082 | ABC | amino acid (glutamine) | probable binding protein component of ABC transporter |
| PA5094 | ABC | glycine betaine/histidine | probable ATP-binding component of ABC transporter |
| PA5095 | ABC | glycine betaine/histidine | probable permease of ABC transporter |
| PA5096 | ABC | glycine betaine/histidine | probable binding protein component of ABC transporter |
| PA5097 | APC | proline | probable amino acid permease |
| PA5099 | NCS1 | cytosine/purines/uracil/thiamine/allantoin | probable transporter |
| PA5101 | ABC | phosphonate | **hypothetical protein** |
| PA5103 | ABC | histidine | **hypothetical protein** |
| PA5121 | MscS | small-conductance mechanosensitive ion channel | **hypothetical protein** |
| PA5137 | ABC | amino acid | **hypothetical protein** |
| PA5138 | ABC | amino acid | **hypothetical protein** |
| PA5139 | ABC | amino acid | **hypothetical protein** |
| PA5152 | ABC | amino acid (lysine/arginine/ornithine/histidine/octopine) | probable ATP-binding component of ABC transporter |
| PA5153 | ABC | amino acid (lysine/arginine/ornithine/histidine/octopine) | probable periplasmic binding protein |
| PA5154 | ABC | amino acid (lysine/arginine/ornithine/histidine/octopine) | probable permease of ABC transporter |
| PA5155 | ABC | amino acid (lysine/arginine/ornithine/histidine/octopine) | probable permease of ABC transporter |
| PA5160 | MFS | multidrug efflux | drug efflux transporter |
| PA5167 | TRAP-T | C4-dicarboxylate | probable c4-dicarboxylate-binding protein |
| PA5168 | TRAP-T | C4-dicarboxylate | probable dicarboxylate transporter |
| PA5169 | TRAP-T | C4-dicarboxylate | probable C4-dicarboxylate transporter |
| PA5170 | APC | arginine/ornithine | arginine/ornithine antiporter |
| PA5207 | PiT | phosphate | probable phosphate transporter |
| PA5216 | ABC | iron(III) | probable permease of ABC iron transporter |
| PA5217 | ABC | iron(III) | probable binding protein component of ABC iron transporter |
| PA5219 | MFS | multidrug efflux | **hypothetical protein** |
| PA5230 | ABC | multidrug | probable permease of ABC transporter |
| PA5231 | ABC | multidrug | probable ATP-binding/permease fusion ABC transporter |
| PA5235 | MFS | glycerol-3-phosphate | glycerol-3-phosphate transporter |
| PA5248 | IRT | lead uptake | **hypothetical protein** |
| PA5249 | RhtB | homoserine/homoserine lactone efflux | **hypothetical protein** |
| PA5251 | MscS | small-conductance mechanosensitive ion channel | **hypothetical protein** |
| PA5252 | ABC | Uup homolog/duplicated ATPase | probable ATP-binding component of ABC transporter |
| PA5268 | MIT | magnesium/cobalt ion | magnesium/cobalt transport protein |
| PA5270 | ABC | amino acid | **hypothetical protein** |
| PA5282 | MFS | multidrug efflux | probable MFS transporter |
| PA5287 | Amt | ammonium | ammonium transporter AmtB |
| PA5291 | BCCT | choline | probable choline transporter |
| PA5294 | MOP | multidrug efflux NorM | **hypothetical protein** |
| PA5311 | MFS |  | probable MFS transporter |
| PA5317 | ABC | dipeptide | probable binding protein component of ABC dipeptide transporter |
| PA5341 | RhtB | amino acid efflux | **hypothetical protein** |
| PA5366 | ABC | phosphate | ATP-binding component of ABC phosphate transporter |
| PA5367 | ABC | phosphate | membrane protein component of ABC phosphate transporter |
| PA5368 | ABC | phosphate | membrane protein component of ABC phosphate transporter |
| PA5369 | ABC | phosphate | **hypothetical protein** |
| PA5370 | MFS | proline/betaine | probable MFS transporter |
| PA5375 | BCCT | choline | choline transporter BetT |
| PA5376 | ABC | glycine betaine/L-proline | probable ATP-binding component of ABC transporter |
| PA5377 | ABC | glycine betaine/L-proline | probable permease of ABC transporter |
| PA5378 | ABC | glycine betaine/L-proline | **hypothetical protein** |
| PA5388 | ABC | glycine betaine/L-proline | **hypothetical protein** |
| PA5434 | HAAAP | tryptophan | tryptophan permease |
| PA5444 | DMT | drug/metabolite | **conserved hypothetical protein** |
| PA5450 | ABC | lipopolysaccharide | ABC subunit of A-band LPS efflux transporter |
| PA5451 | ABC | lipopolysaccharide | membrane subunit of A-band LPS efflux transporter |
| PA5466 | AEC |  | **hypothetical protein** |
| PA5468 | CitMHS | magnesium ion/citrate | probable citrate transporter |
| PA5472 | ABC | amino acid | **hypothetical protein** |
| PA5473 | PNaS | sodium ion/phosphate | **conserved hypothetical protein** |
| PA5476 | MFS | citrate:proton symporter | citrate transporter |
| PA5479 | DAACS | proton:glutamate | proton-glutamate symporter |
| PA5498 | ABC | zinc ion | probable adhesin |
| PA5500 | ABC | zinc ion | zinc transport protein ZnuC |
| PA5501 | ABC | zinc ion | permease of ABC zinc transporter ZnuB |
| PA5503 | ABC | D-methionine | probable ATP-binding component of ABC transporter |
| PA5504 | ABC | D-methionine | probable permease of ABC transporter |
| PA5505 | ABC | D-methionine | probable TonB-dependent receptor |
| PA5510 | APC | amino acid | probable transporter |
| PA5518 | CPA2 | potassium ion efflux | probable potassium efflux transporter |
| PA5529 | CPA2 | sodium ion/proton antiporter | probable sodium/proton antiporter |
| PA5530 | MFS | dicarboxylate (alpha-ketoglutarate) | probable MFS dicarboxylate transporter |
| PA5544 | TRAP-T |  | **conserved hypothetical protein** |
| PA5545 | TRAP-T |  | **conserved hypothetical protein** |
| PA5548 | MFS | multidrug efflux | probable MFS transporter |
| PA5553 | F-ATPase | protons | ATP synthase epsilon chain |
| PA5554 | F-ATPase | protons | ATP synthase beta chain |
| PA5555 | F-ATPase | protons | ATP synthase gamma chain |
| PA5556 | F-ATPase | protons | ATP synthase alpha chain |
| PA5557 | F-ATPase | protons | ATP synthase delta chain |
| PA5558 | F-ATPase | protons | ATP synthase B chain |
| PA5559 | F-ATPase | protons | atp synthase C chain |
| PA5560 | F-ATPase | protons | ATP synthase A chain |
| PA5561 | F-ATPase | protons | ATP synthase protein I |
| PA5568 | Oxa1 | 60 KD inner membrane protein OxaA homolog | **conserved hypothetical protein** |

^a^Transporter family designations according to the TC (transport classification) system ([www.membranetransport.org](http://www.membranetransport.org/), [www.tcdb.org](http://www.tcdb.org/) ).

^b^Annotations derived from the original genome paper (Stover et al. 2000).
