# Supplementary material for: High-Throughput Phenotypic Characterization of Pseudomonas aeruginosa Membrane Transport Genes
Source: PLoS Genet. 2008 Oct 3;4(10):e1000211. doi: 10.1371/journal.pgen.1000211 (PMC2542419; doi:10.1371/journal.pgen.1000211)
Supplement: Table S2 — Pseudomonas aeruginosa PA01 substrate utilization profiles from Biolog phenotype MicroArrays. (1.42 MB DOC) [file pgen.1000211.s002.doc]

**Table S2**. *Pseudomonas aeruginosa* PA01 substrate utilization profiles from Biolog phenotype MicroArrays.

| PM plate | PM  well | Tested substrate | Mode of Action | Substrate utilization^a^ | Growth |
| --- | --- | --- | --- | --- | --- |
| PM01 | H01 | Glycyl-L-Proline | C-Source | 26668 | Positive |
| PM01 | H04 | Tyramine | C-Source | 24222 | Positive |
| PM01 | B12 | L-Glutamic Acid | C-Source | 23878 | Positive |
| PM01 | E01 | L-Glutamine | C-Source | 22571 | Positive |
| PM01 | H06 | L-Lyxose | C-Source | 21993 | Positive |
| PM01 | A08 | L-Proline | C-Source | 21673 | Positive |
| PM01 | H02 | p-Hydroxy Phenyl Acetic Acid | C-Source | 20923 | Positive |
| PM01 | C04 | D-Ribose | C-Source | 20710 | Positive |
| PM01 | H08 | Pyruvic Acid | C-Source | 20157 | Positive |
| PM01 | G05 | L-Alanine | C-Source | 19752 | Positive |
| PM01 | H05 | D-Psicose | C-Source | 19685 | Positive |
| PM01 | D01 | L-Asparagine | C-Source | 19598 | Positive |
| PM01 | G12 | L-Malic Acid | C-Source | 18999 | Positive |
| PM01 | H12 | 2-Aminoethanol | C-Source | 18944 | Positive |
| PM01 | B11 | D-Mannitol | C-Source | 16744 | Positive |
| PM01 | B08 | D-Xylose | C-Source | 16187 | Positive |
| PM01 | F05 | Fumaric Acid | C-Source | 15838 | Positive |
| PM01 | A07 | L-Aspartic Acid | C-Source | 15827 | Positive |
| PM01 | F02 | Citric Acid | C-Source | 14725 | Positive |
| PM01 | A02 | L-Arabinose | C-Source | 14311 | Positive |
| PM01 | C03 | D,L-Malic Acid | C-Source | 12781 | Positive |
| PM01 | B06 | D-Gluconic Acid | C-Source | 12764 | Positive |
| PM01 | C07 | D-Fructose | C-Source | 12379 | Positive |
| PM01 | C09 | D-(+)-Glucose | C-Source | 12219 | Positive |
| PM01 | A03 | N-Acetyl-D-Glucosamine | C-Source | 12057 | Positive |
| PM01 | A09 | D-Alanine | C-Source | 11379 | Positive |
| PM01 | G10 | Methyl Pyruvate | C-Source | 11075 | Positive |
| PM01 | A05 | Succinic Acid | C-Source | 10345 | Positive |
| PM01 | C08 | Acetic Acid | C-Source | 7334 | Marginal |
| PM01 | G11 | D-Malic Acid | C-Source | 7062 | Marginal |
| PM01 | F07 | Propionic Acid | C-Source | 7009 | Marginal |
| PM01 | H07 | Glucuronamide | C-Source | 6787 | Marginal |
| PM01 | D06 | a-Keto-Gutaric Acid | C-Source | 6070 | Marginal |
| PM01 | E04 | D-Fructose-6-Phosphate | C-Source | 5382 | Marginal |
| PM01 | B03 | Glycerol | C-Source | 4879 | Marginal |
| PM01 | D05 | Tween 40 | C-Source | 4762 | Marginal |
| PM01 | B09 | L-Lactic Acid | C-Source | 4598 | Marginal |
| PM01 | C05 | Tween 20 | C-Source | 4562 | Marginal |
| PM01 | B01 | D-Serine | C-Source | 4536 | Marginal |
| PM01 | F06 | Bromo Succinic Acid | C-Source | 4424 | Marginal |
| PM01 | G01 | Glycyl-L-Glutamic Acid | C-Source | 4254 | Marginal |
| PM01 | F12 | Inosine | C-Source | 3809 | Marginal |
| PM01 | G06 | Ala-Gly | C-Source | 3252 | Marginal |
| PM01 | G03 | L-Serine | C-Source | 3041 | Marginal |
| PM01 | D07 | a-Ketobutyric Acid | C-Source | 2763 | Marginal |
| PM01 | E05 | Tween 80 | C-Source | 2637 | Marginal |
| PM01 | E12 | Adenosine | C-Source | 2199 | Marginal |
| PM01 | F01 | Glycyl-L-Aspartic Acid | C-Source | 1802 | Marginal |
| PM01 | F11 | D-Cellobiose | C-Source | 1274 | Marginal |
| PM01 | G02 | Tricarballylic Acid | C-Source | 1045 | Marginal |
| PM01 | G09 | Mono Methyl Succinate | C-Source | 998 | None |
| PM01 | C06 | L-Rhamnose | C-Source | 886 | None |
| PM01 | A10 | D-Trehalose | C-Source | 839 | None |
| PM01 | D12 | Uridine | C-Source | 660 | None |
| PM01 | G08 | N-Acetyl-D-Mannosamine | C-Source | 646 | None |
| PM01 | H10 | D-Galacturonic Acid | C-Source | 423 | None |
| PM01 | A06 | D-Galactose | C-Source | 349 | None |
| PM01 | D04 | 1,2-Propanediol | C-Source | 283 | None |
| PM01 | E11 | 2'-Deoxy Adenosine | C-Source | 283 | None |
| PM01 | E07 | D,L-a-Hydroxy-Butyric Acid | C-Source | 159 | None |
| PM01 | H03 | M-Hydroxy Phenyl Acetic Acid | C-Source | 133 | None |
| PM01 | G07 | Acetoacetic Acid | C-Source | 94 | None |
| PM01 | C12 | Thymidine | C-Source | 88 | None |
| PM01 | E06 | a-Hydroxy Glutaric Acid-g-Lactone | C-Source | 85 | None |
| PM01 | D11 | Sucrose | C-Source | 43 | None |
| PM01 | C10 | Maltose | C-Source | 30 | None |
| PM01 | C11 | D-Melibiose | C-Source | 18 | None |
| PM01 | E02 | m-Tartaric Acid | C-Source | 11 | None |
| PM01 | D08 | a-Methyl-D-Galactoside | C-Source | 6 | None |
| PM01 | G04 | L-Threonine | C-Source | 5 | None |
| PM01 | A04 | D-Saccharic Acid | C-Source | 4 | None |
| PM01 | B04 | L-Fucose | C-Source | 4 | None |
| PM01 | D09 | a-D-Lactose | C-Source | 4 | None |
| PM01 | A12 | Dulcitol | C-Source | 2 | None |
| PM01 | E10 | Maltotriose | C-Source | 2 | None |
| PM01 | B05 | D-Glucuronic Acid | C-Source | 1 | None |
| PM01 | B07 | D,L-a-Glycerol Phosphate | C-Source | 1 | None |
| PM01 | D10 | Lactulose | C-Source | 1 | None |
| PM01 | A01 | Background | C-Source | 0 | None |
| PM01 | A11 | D-Mannose | C-Source | 0 | None |
| PM01 | B02 | D-Sorbitol | C-Source | 0 | None |
| PM01 | B10 | Formic Acid | C-Source | 0 | None |
| PM01 | C01 | D-Glucose-6-Phosphate | C-Source | 0 | None |
| PM01 | C02 | D-Galactonic Acid-g-Lactone | C-Source | 0 | None |
| PM01 | D02 | D-Aspartic Acid | C-Source | 0 | None |
| PM01 | D03 | D-Glucosaminic Acid | C-Source | 0 | None |
| PM01 | E03 | D-Glucose-1-Phosphate | C-Source | 0 | None |
| PM01 | E08 | b-Methyl-D-Glucoside | C-Source | 0 | None |
| PM01 | E09 | Adonitol | C-Source | 0 | None |
| PM01 | F03 | m-Inositol | C-Source | 0 | None |
| PM01 | F04 | D-Threonine | C-Source | 0 | None |
| PM01 | F08 | Mucic Acid | C-Source | 0 | None |
| PM01 | F09 | Glycolic Acid | C-Source | 0 | None |
| PM01 | F10 | Glyoxylic Acid | C-Source | 0 | None |
| PM01 | H09 | L-Galactonic Acid-g-Lactone | C-Source | 0 | None |
| PM01 | H11 | b-Phenylethylamine | C-Source | 0 | None |
| PM02 | H09 | Dihydroxy Acetone | C-Source | 29119 | Positive |
| PM02 | H07 | D.L-Octopamine | C-Source | 26282 | Positive |
| PM02 | H08 | Putrescine | C-Source | 24835 | Positive |
| PM02 | H03 | L-Pyroglutamic Acid | C-Source | 23992 | Positive |
| PM02 | D10 | g-Amino Butyric Acid | C-Source | 22562 | Positive |
| PM02 | E05 | D-Glucosamine | C-Source | 21809 | Positive |
| PM02 | H01 | L-Ornithine | C-Source | 19433 | Positive |
| PM02 | G08 | Hydroxy-L-Proline | C-Source | 19260 | Positive |
| PM02 | G03 | N-Acetyl-L-Glutamic Acid | C-Source | 18747 | Positive |
| PM02 | E12 | 5-Keto-D-Gluconic Acid | C-Source | 18273 | Positive |
| PM02 | F09 | Sorbic acid | C-Source | 17796 | Positive |
| PM02 | G04 | L-Arginine | C-Source | 16848 | Positive |
| PM02 | G06 | L-Histidine | C-Source | 16311 | Positive |
| PM02 | H11 | Diacetyl | C-Source | 14348 | Positive |
| PM02 | B09 | 2-Deoxy-D-Ribose | C-Source | 13525 | Positive |
| PM02 | B05 | D-Arabinose | C-Source | 12620 | Positive |
| PM02 | F06 | Quinic Acid | C-Source | 12403 | Positive |
| PM02 | H02 | L-Phenylalanine | C-Source | 11859 | Positive |
| PM02 | F05 | Oxalomalic Acid | C-Source | 11825 | Positive |
| PM02 | B12 | 3-0-b-D-Galacto-pyranosyl-D-Arabinose | C-Source | 11813 | Positive |
| PM02 | G09 | L-Isoleucine | C-Source | 11680 | Positive |
| PM02 | D11 | d-Amino Valeric Acid | C-Source | 11281 | Positive |
| PM02 | H05 | D,L-Carnitine | C-Source | 9966 | Marginal |
| PM02 | E04 | Citramalic Acid | C-Source | 9321 | Marginal |
| PM02 | E01 | Capric Acid | C-Source | 9108 | Marginal |
| PM02 | E11 | Itaconic Acid | C-Source | 8801 | Marginal |
| PM02 | E02 | Caproic Acid | C-Source | 8226 | Marginal |
| PM02 | E10 | 2-Oxovaleric acid | C-Source | 7678 | Marginal |
| PM02 | D06 | D-Tagatose | C-Source | 7364 | Marginal |
| PM02 | E07 | 4-Hydroxy Benzoic Acid Sodium | C-Source | 6365 | Marginal |
| PM02 | F02 | Malonic Acid | C-Source | 5117 | Marginal |
| PM02 | D12 | Butyric Acid | C-Source | 4409 | Marginal |
| PM02 | A11 | Mannan | C-Source | 4088 | Marginal |
| PM02 | C12 | Palatinose | C-Source | 4056 | Marginal |
| PM02 | F01 | D-Lactic Acid Methyl Ester | C-Source | 3434 | Marginal |
| PM02 | E08 | b-Hydroxy Butyric Acid | C-Source | 3369 | Marginal |
| PM02 | B08 | Arbutin | C-Source | 1950 | Marginal |
| PM02 | A12 | Pectin | C-Source | 1849 | Marginal |
| PM02 | A09 | Inulin | C-Source | 1420 | Marginal |
| PM02 | C01 | Gentiobiose | C-Source | 1229 | Marginal |
| PM02 | H12 | 3-Hydroxy 2-Butanone | C-Source | 1167 | Marginal |
| PM02 | D09 | N-Acetyl-D-glucosaminitol | C-Source | 1067 | Marginal |
| PM02 | C09 | b-Methyl-D-Glucuronic Acid | C-Source | 925 | None |
| PM02 | H10 | 2,3-Butanediol | C-Source | 910 | None |
| PM02 | D01 | D-Raffinose | C-Source | 764 | None |
| PM02 | F08 | Sebacic Acid | C-Source | 682 | None |
| PM02 | D04 | L-Sorbose | C-Source | 680 | None |
| PM02 | F04 | Oxolic Acid | C-Source | 526 | None |
| PM02 | B03 | b-D-Allose | C-Source | 523 | None |
| PM02 | G10 | L-Leucine | C-Source | 333 | None |
| PM02 | G11 | L-Lysine | C-Source | 297 | None |
| PM02 | H04 | L-Valine | C-Source | 268 | None |
| PM02 | F10 | Succinamic Acid | C-Source | 248 | None |
| PM02 | C10 | a-Methyl-D-Mannoside | C-Source | 238 | None |
| PM02 | F12 | L-Tartaric Acid | C-Source | 180 | None |
| PM02 | G01 | Acetamide | C-Source | 109 | None |
| PM02 | G12 | L-Methionine | C-Source | 54 | None |
| PM02 | B06 | D-Arabitol | C-Source | 11 | None |
| PM02 | A01 | Background | C-Source | 0 | None |
| PM02 | B11 | D-Fucose | C-Source | -63 | None |
| PM02 | B01 | N-Acetyl-D-Galactosamine | C-Source | -64 | None |
| PM02 | A06 | Dextrin | C-Source | -84 | None |
| PM02 | F11 | D-Tartaric Acid | C-Source | -85 | None |
| PM02 | D05 | Stachyose | C-Source | -86 | None |
| PM02 | E03 | Citraconic Acid | C-Source | -92 | None |
| PM02 | C08 | 3-Methyl Glucose | C-Source | -99 | None |
| PM02 | B02 | N-Acetyl-Neuraminic Acid | C-Source | -106 | None |
| PM02 | G07 | L-Homoserine | C-Source | -107 | None |
| PM02 | G02 | L-Alaninamide | C-Source | -109 | None |
| PM02 | G05 | Glycine | C-Source | -109 | None |
| PM02 | D02 | Salicin | C-Source | -113 | None |
| PM02 | H06 | Sec-Butylamine | C-Source | -115 | None |
| PM02 | A08 | Glycogen | C-Source | -116 | None |
| PM02 | C05 | Maltitol | C-Source | -116 | None |
| PM02 | F07 | D-Ribono-1,4-Lactone | C-Source | -116 | None |
| PM02 | F03 | Melibionic Acid | C-Source | -119 | None |
| PM02 | C11 | b-Methyl-D-Xyloside | C-Source | -120 | None |
| PM02 | A10 | Laminarin | C-Source | -122 | None |
| PM02 | D08 | Xylitol | C-Source | -122 | None |
| PM02 | E06 | 2-Hydroxybenzoic acid | C-Source | -123 | None |
| PM02 | A07 | Gelatin | C-Source | -124 | None |
| PM02 | B10 | i-Erythritol | C-Source | -124 | None |
| PM02 | A05 | g-Cyclodextrin | C-Source | -125 | None |
| PM02 | A03 | a-Cyclodextrin | C-Source | -131 | None |
| PM02 | C02 | L-Glucose | C-Source | -135 | None |
| PM02 | C06 | a-Methyl-D-Galactoside | C-Source | -136 | None |
| PM02 | D03 | Sedoheptulosan | C-Source | -136 | None |
| PM02 | E09 | g-Hydroxy Butyric Acid | C-Source | -136 | None |
| PM02 | A04 | b-Cyclodextrin | C-Source | -137 | None |
| PM02 | B04 | D-Amygdalin | C-Source | -137 | None |
| PM02 | C03 | Lactitol | C-Source | -137 | None |
| PM02 | D07 | Turanose | C-Source | -137 | None |
| PM02 | B07 | L-Arabitol | C-Source | -138 | None |
| PM02 | C07 | b-Methyl-D-Galactoside | C-Source | -140 | None |
| PM02 | A02 | Chondroitin Sulfate C | C-Source | -141 | None |
| PM02 | C04 | D-Melezitose | C-Source | -141 | None |
| PM03 | C01 | L-Tyrosine | N-Source | 23625 | Positive |
| PM03 | D12 | Agmatine | N-Source | 23403 | Positive |
| PM03 | B01 | L-Glutamine | N-Source | 20998 | Positive |
| PM03 | E01 | Histamine | N-Source | 20644 | Positive |
| PM03 | H02 | Ala-Gln | N-Source | 20614 | Positive |
| PM03 | G01 | Xanthine | N-Source | 19947 | Positive |
| PM03 | G03 | Uric Acid | N-Source | 19898 | Positive |
| PM03 | H03 | Ala-Glu | N-Source | 19828 | Positive |
| PM03 | A08 | L-Arginine | N-Source | 19798 | Positive |
| PM03 | H08 | Gly-Asn | N-Source | 19686 | Positive |
| PM03 | D11 | Putrescine | N-Source | 19447 | Positive |
| PM03 | C12 | L-Ornithine | N-Source | 19383 | Positive |
| PM03 | H05 | Ala-His | N-Source | 19179 | Positive |
| PM03 | H09 | Gly-Gln | N-Source | 18504 | Positive |
| PM03 | G05 | Allantoin | N-Source | 18396 | Positive |
| PM03 | A09 | L-Asparagine | N-Source | 17953 | Positive |
| PM03 | B03 | L-Histidine | N-Source | 17605 | Positive |
| PM03 | H04 | Ala-Gly | N-Source | 17248 | Positive |
| PM03 | B12 | L-Tryptophan | N-Source | 17217 | Positive |
| PM03 | H06 | Ala-Leu | N-Source | 16818 | Positive |
| PM03 | G08 | g-Amino Butyric Acid | N-Source | 16816 | Positive |
| PM03 | A10 | L-Aspartic Acid | N-Source | 16714 | Positive |
| PM03 | D09 | Ethanolamine | N-Source | 16527 | Positive |
| PM03 | E04 | Acetamide | N-Source | 16398 | Positive |
| PM03 | E10 | D-Mannosamine | N-Source | 16353 | Positive |
| PM03 | A07 | L-Alanine | N-Source | 16032 | Positive |
| PM03 | D03 | L-Pyroglutamic Acid | N-Source | 15974 | Positive |
| PM03 | A12 | L-Glutamic Acid | N-Source | 15812 | Positive |
| PM03 | A05 | Urea | N-Source | 15802 | Positive |
| PM03 | B02 | Glycine | N-Source | 15625 | Positive |
| PM03 | C06 | D-Glutamic Acid | N-Source | 15601 | Positive |
| PM03 | H01 | Ala-Asp | N-Source | 15554 | Positive |
| PM03 | B09 | L-Proline | N-Source | 15388 | Positive |
| PM03 | B08 | L-Phenylalanine | N-Source | 15345 | Positive |
| PM03 | F04 | Cytidine | N-Source | 15319 | Positive |
| PM03 | E03 | Tyramine | N-Source | 15007 | Positive |
| PM03 | A02 | Ammonia | N-Source | 14769 | Positive |
| PM03 | F07 | Guanosine | N-Source | 14723 | Positive |
| PM03 | F05 | Cytosine | N-Source | 14576 | Positive |
| PM03 | C03 | D-Alanine | N-Source | 14172 | Positive |
| PM03 | F02 | Adenine | N-Source | 14164 | Positive |
| PM03 | F12 | Inosine | N-Source | 13652 | Positive |
| PM03 | B04 | L-Isoleucine | N-Source | 13581 | Positive |
| PM03 | B10 | L-Serine | N-Source | 13303 | Positive |
| PM03 | G12 | a-Amino-N-Valeric Acid | N-Source | 13283 | Positive |
| PM03 | H07 | Ala-Thr | N-Source | 13256 | Positive |
| PM03 | G11 | d-Amino-N-Valeric Acid | N-Source | 13040 | Positive |
| PM03 | C02 | L-Valine | N-Source | 12891 | Positive |
| PM03 | F06 | Guanine | N-Source | 12640 | Positive |
| PM03 | H10 | Gly-Glu | N-Source | 12565 | Positive |
| PM03 | G04 | Alloxan | N-Source | 12041 | Positive |
| PM03 | C04 | D-Asparagine | N-Source | 12021 | Positive |
| PM03 | D01 | N-Acetyl-L-Glutamic Acid | N-Source | 11916 | Positive |
| PM03 | E11 | N-Acetyl-D-Glucosamine | N-Source | 11780 | Positive |
| PM03 | B05 | L-Leucine | N-Source | 11593 | Positive |
| PM03 | G06 | Parabanic Acid | N-Source | 11526 | Positive |
| PM03 | F01 | N-Acetyl-D-Mannosamine | N-Source | 11293 | Positive |
| PM03 | E12 | N-Acetyl-D-Galactosamine | N-Source | 11292 | Positive |
| PM03 | F10 | Uracil | N-Source | 11291 | Positive |
| PM03 | F03 | Adenosine | N-Source | 10893 | Positive |
| PM03 | G02 | Xanthosine | N-Source | 10862 | Positive |
| PM03 | F11 | Uridine | N-Source | 10830 | Positive |
| PM03 | A04 | Sodium Nitrate | N-Source | 10275 | Positive |
| PM03 | F08 | Thymine | N-Source | 10005 | Positive |
| PM03 | E06 | Glucuronamide | N-Source | 8276 | Marginal |
| PM03 | A11 | L-Cysteine | N-Source | 8224 | Marginal |
| PM03 | A03 | Nitrite | N-Source | 7963 | Marginal |
| PM03 | G07 | D,L-a-Amino-N-Butyric Acid | N-Source | 7739 | Marginal |
| PM03 | E09 | D-Galactosamine | N-Source | 6980 | Marginal |
| PM03 | B06 | L-Lysine | N-Source | 6595 | Marginal |
| PM03 | A06 | Biuret | N-Source | 5476 | Marginal |
| PM03 | B11 | L-Threonine | N-Source | 5427 | Marginal |
| PM03 | E07 | D,L-Lactamide | N-Source | 5419 | Marginal |
| PM03 | C09 | D-Valine | N-Source | 5259 | Marginal |
| PM03 | E05 | Formamide | N-Source | 4802 | Marginal |
| PM03 | D02 | N-Phthaloyl-L-Glutamic Acid | N-Source | 4662 | Marginal |
| PM03 | H12 | Met-Ala | N-Source | 4586 | Marginal |
| PM03 | H11 | Gly-Met | N-Source | 4234 | Marginal |
| PM03 | C08 | D-Serine | N-Source | 3758 | Marginal |
| PM03 | C10 | L-Citrulline | N-Source | 3311 | Marginal |
| PM03 | B07 | L-Methionine | N-Source | 2511 | Marginal |
| PM03 | C07 | D-Lysine | N-Source | 1402 | Marginal |
| PM03 | A01 | Background | N-Source | 0 | None |
| PM03 | D10 | Ethylenediamine | N-Source | -557 | None |
| PM03 | G09 | e-Amino-N-Caproic Acid | N-Source | -575 | None |
| PM03 | E08 | D-Glucosamine | N-Source | -1238 | None |
| PM03 | D08 | Ethylamine | N-Source | -2016 | None |
| PM03 | C05 | D-Aspartic Acid | N-Source | -2089 | None |
| PM03 | E02 | b-Phenylethylamine | N-Source | -2113 | None |
| PM03 | G10 | D,L-a-Amino- Caprylic Acid | N-Source | -2202 | None |
| PM03 | D06 | N-Amylamine | N-Source | -2253 | None |
| PM03 | F09 | Thymidine | N-Source | -2268 | None |
| PM03 | D05 | Methylamine | N-Source | -2333 | None |
| PM03 | D07 | N-Butylamine | N-Source | -2387 | None |
| PM03 | C11 | L-Homoserine | N-Source | -2396 | None |
| PM03 | D04 | Hydroxylamine | N-Source | -2396 | None |
| PM04 | B01 | Thiophosphate | P-Source | 9409 | Marginal |
| PM04 | B02 | Dithiophosphate | P-Source | 8448 | Marginal |
| PM04 | E11 | Inositol Hexaphosphate | P-Source | 7872 | Marginal |
| PM04 | D03 | Cysteamine-S-Phosphate | P-Source | 4205 | Marginal |
| PM04 | A03 | Sodium pyrophosphate | P-Source | 4187 | Marginal |
| PM04 | E06 | Phosphono Acetic Acid | P-Source | 1600 | Marginal |
| PM04 | A07 | Hypophosphite | P-Source | 1583 | Marginal |
| PM04 | C06 | D-Glucosamine-6-Phosphate | P-Source | 1334 | Marginal |
| PM04 | A06 | Triethyl Phosphate | P-Source | 853 | None |
| PM04 | A01 | Background | P-Source | 0 | None |
| PM04 | E12 | Thymidine 3',5'- cyclic monophosphate | P-Source | -272 | None |
| PM04 | B12 | Guanosine- 3',5'-cyclic monophosphate | P-Source | -606 | None |
| PM04 | E02 | O-Phospho-L-Tyrosine | P-Source | -980 | None |
| PM04 | D12 | Uridine- 3',5'- cyclic monophosphate | P-Source | -1251 | None |
| PM04 | E01 | O-Phospho-D-Tyrosine | P-Source | -1611 | None |
| PM04 | A05 | Tripolyphosphate | P-Source | -2403 | None |
| PM04 | E04 | Phosphoryl Choline | P-Source | -2607 | None |
| PM04 | C12 | Cytidine- 3',5'-cyclic monophosphate | P-Source | -2609 | None |
| PM04 | C05 | 2-Deoxy-D-Glucose 6-Phosphate | P-Source | -2820 | None |
| PM04 | B08 | Guanosine- 2'-monophosphate | P-Source | -3110 | None |
| PM04 | A10 | Adenosine- 5'-monophosphate | P-Source | -3233 | None |
| PM04 | A02 | Phosphate | P-Source | -3284 | None |
| PM04 | A12 | Adenosine- 3',5'-cyclic monophosphate | P-Source | -3511 | None |
| PM04 | A04 | Trimetaphosphate | P-Source | -3541 | None |
| PM04 | E07 | 2-Aminoethyl Phosphonic Acid | P-Source | -3755 | None |
| PM04 | D01 | D-Mannose-1-Phosphate | P-Source | -3778 | None |
| PM04 | A11 | Adenosine- 2',3'-cyclic monophosphate | P-Source | -3816 | None |
| PM04 | B03 | D,L-a-Glycerol Phosphate | P-Source | -3940 | None |
| PM04 | E10 | Thymidine- 5'-monophosphate | P-Source | -4024 | None |
| PM04 | C07 | 6-Phospho-Gluconic Acid | P-Source | -4042 | None |
| PM04 | C11 | Cytidine- 2',3'-cyclic monophosphate | P-Source | -4043 | None |
| PM04 | A08 | Adenosine- 2'-monophosphate | P-Source | -4087 | None |
| PM04 | B10 | Guanosine- 5'-monophosphate | P-Source | -4096 | None |
| PM04 | C01 | Phosphoenol Pyruvate | P-Source | -4153 | None |
| PM04 | D06 | O-Phospho-L-Serine | P-Source | -4206 | None |
| PM04 | E05 | O-Phosphoryl-Ethanolamine | P-Source | -4235 | None |
| PM04 | B11 | Guanosine- 2',3'-cyclic monophosphate | P-Source | -4277 | None |
| PM04 | A09 | Adenosine- 3'-monophosphate | P-Source | -4329 | None |
| PM04 | D04 | Phospho-L-Arginine | P-Source | -4448 | None |
| PM04 | B06 | D-2-Phospho-Glyceric Acid | P-Source | -4470 | None |
| PM04 | D07 | O-Phospho-L-Threonine | P-Source | -4476 | None |
| PM04 | C04 | D-Glucose-6-Phosphate | P-Source | -4643 | None |
| PM04 | D02 | D-Mannose-6-Phosphate | P-Source | -4679 | None |
| PM04 | B04 | b-Glycerol Phosphate | P-Source | -4719 | None |
| PM04 | E03 | Phosphocreatine | P-Source | -4763 | None |
| PM04 | E09 | Thymidine- 3'-monophosphate | P-Source | -4771 | None |
| PM04 | D08 | Uridine- 2'- monophosphate | P-Source | -4778 | None |
| PM04 | C03 | D-Glucose-1-Phosphate | P-Source | -4858 | None |
| PM04 | B09 | Guanosine- 3'-monophosphate | P-Source | -4930 | None |
| PM04 | D11 | Uridine- 2',3'- cyclic monophosphate | P-Source | -5068 | None |
| PM04 | D10 | Uridine- 5'- monophosphate | P-Source | -5214 | None |
| PM04 | D05 | O-Phospho-D-Serine | P-Source | -5308 | None |
| PM04 | C08 | Cytidine- 2'- monophosphate | P-Source | -5320 | None |
| PM04 | B05 | L-a -Phosphatidyl- D,L-Glycerol | P-Source | -5366 | None |
| PM04 | C10 | Cytidine- 5'- monophosphate | P-Source | -5400 | None |
| PM04 | C02 | Phospho- Glycolic Acid | P-Source | -5528 | None |
| PM04 | C09 | Cytidine- 3'- monophosphate | P-Source | -5552 | None |
| PM04 | B07 | D-3-Phospho-Glyceric Acid | P-Source | -5555 | None |
| PM04 | D09 | Uridine- 3'- monophosphate | P-Source | -5642 | None |
| PM04 | E08 | Methylene Diphosphonic Acid | P-Source | -9875 | None |
| PM04 | H06 | Taurine | S-Source | 17132 | Positive |
| PM04 | H07 | Hypotaurine | S-Source | 16934 | Positive |
| PM04 | F12 | L-Cysteine Sulfinic Acid | S-Source | 16167 | Positive |
| PM04 | F03 | Thiosulfate | S-Source | 16115 | Positive |
| PM04 | G09 | Gly-Met | S-Source | 16038 | Positive |
| PM04 | F04 | Tetrathionate | S-Source | 15855 | Positive |
| PM04 | F07 | L-Cysteine | S-Source | 15813 | Positive |
| PM04 | G12 | L-Methionine Sulfone | S-Source | 15651 | Positive |
| PM04 | H10 | 2-Hydroxyethane Sulfonic Acid | S-Source | 14997 | Positive |
| PM04 | G11 | L- Methionine Sulfoxide | S-Source | 14970 | Positive |
| PM04 | H09 | Butane Sulfonic Acid | S-Source | 14930 | Positive |
| PM04 | F02 | Sulfate | S-Source | 14924 | Positive |
| PM04 | H11 | Methane Sulfonic Acid | S-Source | 14665 | Positive |
| PM04 | G08 | D-Methionine | S-Source | 14486 | Positive |
| PM04 | G02 | S-Methyl-L-Cysteine | S-Source | 14402 | Positive |
| PM04 | G07 | L-Methionine | S-Source | 13705 | Positive |
| PM04 | H04 | D,L-Lipoamide | S-Source | 13082 | Positive |
| PM04 | G03 | Cystathionine | S-Source | 12873 | Positive |
| PM04 | F06 | Dithiophosphate | S-Source | 12693 | Positive |
| PM04 | G10 | N-Acetyl-D,L-Methionine | S-Source | 12053 | Positive |
| PM04 | H02 | Thiourea | S-Source | 11902 | Positive |
| PM04 | F05 | Thiophosphate | S-Source | 10486 | Positive |
| PM04 | F11 | Cysteamine | S-Source | 9441 | Marginal |
| PM04 | G04 | Lanthionine | S-Source | 7412 | Marginal |
| PM04 | H01 | L-Djenkolic Acid | S-Source | 5836 | Marginal |
| PM04 | F09 | L-Cysteinyl-Glycine | S-Source | 5096 | Marginal |
| PM04 | G06 | D,L-Ethionine | S-Source | 4939 | Marginal |
| PM04 | F10 | L-Cysteic Acid | S-Source | 3867 | Marginal |
| PM04 | H05 | Taurocholic Acid | S-Source | 3548 | Marginal |
| PM04 | H12 | Tetramethylene Sulfone | S-Source | 2581 | Marginal |
| PM04 | H03 | 1-Thio-b-D-Glucose | S-Source | 2350 | Marginal |
| PM04 | F08 | D-Cysteine | S-Source | 2305 | Marginal |
| PM04 | G05 | Glutathione | S-Source | 2154 | Marginal |
| PM04 | H08 | p-Amino Benzene Sulfonic Acid | S-Source | 2091 | Marginal |
| PM04 | G01 | N-Acetyl-L-Cysteine | S-Source | 1012 | Marginal |
| PM04 | F01 | Background | S-Source | 0 | None |
| PM05 | H01 | Butyric Acid | nutrient stimulation | 3289 |  |
| PM05 | G12 | m-Inositol | nutrient stimulation | 2750 |  |
| PM05 | F12 | Thymidine | nutrient stimulation | 2712 |  |
| PM05 | D12 | 2'-Deoxy Cytidine | nutrient stimulation | 2529 |  |
| PM05 | G01 | Oxaloacetic Acid | nutrient stimulation | 2439 |  |
| PM05 | H07 | DL-Carnitine | nutrient stimulation | 2409 |  |
| PM05 | H03 | a-Ketobutyric Acid | nutrient stimulation | 2294 |  |
| PM05 | E12 | 2'-Deoxyuridine | nutrient stimulation | 2288 |  |
| PM05 | H10 | Tween 40 | nutrient stimulation | 1982 |  |
| PM05 | A02 | Positive Control | nutrient stimulation | 1853 |  |
| PM05 | H06 | DL-Mevalonic Acid | nutrient stimulation | 1846 |  |
| PM05 | H04 | Caprylic Acid | nutrient stimulation | 1838 |  |
| PM05 | B12 | 2'-Deoxy Guanosine | nutrient stimulation | 1715 |  |
| PM05 | C12 | 2'-DeoxyInosine | nutrient stimulation | 1572 |  |
| PM05 | H08 | Choline | nutrient stimulation | 1551 |  |
| PM05 | E01 | Putrescine | nutrient stimulation | 1490 |  |
| PM05 | B01 | L-Glutamine | nutrient stimulation | 1471 |  |
| PM05 | H11 | Tween 60 | nutrient stimulation | 1270 |  |
| PM05 | H12 | Tween 80 | nutrient stimulation | 1181 |  |
| PM05 | H05 | DL-a-Lipoic Acid (oxidized form) | nutrient stimulation | 1124 |  |
| PM05 | H09 | Tween 20 | nutrient stimulation | 1062 |  |
| PM05 | H02 | DL-a-Hydroxy-Butyric Acid | nutrient stimulation | 869 |  |
| PM05 | A06 | L-Aspartic Acid | nutrient stimulation | 720 |  |
| PM05 | D01 | L-Ornithine | nutrient stimulation | 495 |  |
| PM05 | G11 | Menadione | nutrient stimulation | 422 |  |
| PM05 | G03 | Cyano-Cobalamine | nutrient stimulation | 394 |  |
| PM05 | F01 | Quinolinic Acid | nutrient stimulation | 388 |  |
| PM05 | F11 | Glutathione (reduced form) | nutrient stimulation | 301 |  |
| PM05 | G04 | p-Amino-Benzoic Acid | nutrient stimulation | 253 |  |
| PM05 | F03 | Nicotinamide | nutrient stimulation | 194 |  |
| PM05 | A12 | 2'-Deoxy Adenosine | nutrient stimulation | 148 |  |
| PM05 | A01 | None Control | nutrient stimulation | 0 |  |
| PM05 | F04 | b-Nicotinamide Adenine Dinucleotide | nutrient stimulation | -12 |  |
| PM05 | F02 | Nicotinic Acid | nutrient stimulation | -65 |  |
| PM05 | G02 | D-Biotin | nutrient stimulation | -144 |  |
| PM05 | E02 | Spermidine | nutrient stimulation | -146 |  |
| PM05 | E04 | Pyridoxine | nutrient stimulation | -181 |  |
| PM05 | G07 | Thiamine | nutrient stimulation | -202 |  |
| PM05 | G06 | Inosine + Thiamine | nutrient stimulation | -217 |  |
| PM05 | G05 | Folic Acid | nutrient stimulation | -236 |  |
| PM05 | A10 | Adenine | nutrient stimulation | -249 |  |
| PM05 | C01 | L-Proline | nutrient stimulation | -261 |  |
| PM05 | D03 | Chorismic Acid | nutrient stimulation | -301 |  |
| PM05 | G08 | Thiamine Pyrophosphate | nutrient stimulation | -331 |  |
| PM05 | G10 | Pyrrolo-Quinoline Quinone | nutrient stimulation | -349 |  |
| PM05 | G09 | Riboflavin | nutrient stimulation | -391 |  |
| PM05 | D10 | Cytosine | nutrient stimulation | -396 |  |
| PM05 | E09 | Orotic Acid | nutrient stimulation | -418 |  |
| PM05 | C09 | (5) 4-Amino-Imidazole-4(5)-Carboxamide | nutrient stimulation | -423 |  |
| PM05 | F10 | Thymine | nutrient stimulation | -442 |  |
| PM05 | D06 | D-Alanine | nutrient stimulation | -480 |  |
| PM05 | B06 | L-Lysine | nutrient stimulation | -482 |  |
| PM05 | D02 | L-Citrulline | nutrient stimulation | -489 |  |
| PM05 | F05 | d-Amino-Levulinic Acid | nutrient stimulation | -495 |  |
| PM05 | E10 | Uracil | nutrient stimulation | -532 |  |
| PM05 | F06 | Hematin | nutrient stimulation | -542 |  |
| PM05 | C04 | L-Tryptophan | nutrient stimulation | -555 |  |
| PM05 | C05 | L-Tyrosine | nutrient stimulation | -621 |  |
| PM05 | A04 | L-Arginine | nutrient stimulation | -655 |  |
| PM05 | C02 | L-Serine | nutrient stimulation | -684 |  |
| PM05 | E07 | b-Alanine | nutrient stimulation | -690 |  |
| PM05 | E06 | Pyridoxamine | nutrient stimulation | -691 |  |
| PM05 | A03 | L-Alanine | nutrient stimulation | -750 |  |
| PM05 | E11 | Uridine | nutrient stimulation | -767 |  |
| PM05 | B02 | Glycine | nutrient stimulation | -794 |  |
| PM05 | E05 | Pyridoxal | nutrient stimulation | -819 |  |
| PM05 | B04 | L-Isoleucine | nutrient stimulation | -823 |  |
| PM05 | F09 | N-Acetyl D-Glucosamine | nutrient stimulation | -824 |  |
| PM05 | D04 | (-)Shikimic Acid | nutrient stimulation | -827 |  |
| PM05 | C07 | L-Isoleucine +L-Valine | nutrient stimulation | -833 |  |
| PM05 | F08 | D-(+)-Glucose | nutrient stimulation | -836 |  |
| PM05 | F07 | Deferoxamine Mesylate | nutrient stimulation | -846 |  |
| PM05 | C10 | Hypoxanthine | nutrient stimulation | -856 |  |
| PM05 | A11 | Adenosine | nutrient stimulation | -870 |  |
| PM05 | C03 | L-Threonine | nutrient stimulation | -954 |  |
| PM05 | B10 | Guanine | nutrient stimulation | -961 |  |
| PM05 | A05 | L-Asparagine | nutrient stimulation | -984 |  |
| PM05 | C06 | L-Valine | nutrient stimulation | -1094 |  |
| PM05 | D08 | D-Glutamic Acid | nutrient stimulation | -1115 |  |
| PM05 | A07 | L-Cysteine | nutrient stimulation | -1148 |  |
| PM05 | D05 | L-Homoserine Lactone | nutrient stimulation | -1183 |  |
| PM05 | B08 | L-Phenylalanine | nutrient stimulation | -1203 |  |
| PM05 | D11 | Cytidine | nutrient stimulation | -1238 |  |
| PM05 | A08 | L-Glutamic Acid | nutrient stimulation | -1242 |  |
| PM05 | C11 | Inosine | nutrient stimulation | -1312 |  |
| PM05 | E03 | Spermine | nutrient stimulation | -1335 |  |
| PM05 | D07 | D-Aspartic Acid | nutrient stimulation | -1400 |  |
| PM05 | A09 | Adenosine-3',5'-cyclic monophosphate | nutrient stimulation | -1424 |  |
| PM05 | C08 | Trans-4-Hydroxy L-Proline | nutrient stimulation | -1452 |  |
| PM05 | E08 | D-Pantothenic Acid | nutrient stimulation | -1484 |  |
| PM05 | B11 | Guanosine | nutrient stimulation | -1507 |  |
| PM05 | B03 | L-Histidine | nutrient stimulation | -1530 |  |
| PM05 | B09 | Guanosine-3',5'-cyclic monophosphate | nutrient stimulation | -1555 |  |
| PM05 | B05 | L-Leucine | nutrient stimulation | -1619 |  |
| PM05 | B07 | L-Methionine | nutrient stimulation | -1724 |  |
| PM05 | D09 | DL-Diamino-Pimelic Acid | nutrient stimulation | -1823 |  |
| PM06 | B12 | Arg-Lys | N-Source | 27001 | Positive |
| PM06 | H05 | Leu-Arg | N-Source | 25336 | Positive |
| PM06 | C05 | Arg-Tyr | N-Source | 25226 | Positive |
| PM06 | C04 | Arg-Trp | N-Source | 22703 | Positive |
| PM06 | B06 | Arg-Arg | N-Source | 22434 | Positive |
| PM06 | C02 | Arg-Phe | N-Source | 22402 | Positive |
| PM06 | A04 | Ala-Arg | N-Source | 21610 | Positive |
| PM06 | B11 | Arg-Leu | N-Source | 21478 | Positive |
| PM06 | C01 | Arg-Met | N-Source | 21385 | Positive |
| PM06 | A02 | Background | N-Source | 21312 | Positive |
| PM06 | C06 | Arg-Val | N-Source | 21139 | Positive |
| PM06 | E03 | Gly-Arg | N-Source | 21082 | Positive |
| PM06 | B04 | Ala-Tyr | N-Source | 20560 | Positive |
| PM06 | B10 | Arg-Ile | N-Source | 20460 | Positive |
| PM06 | G04 | Ile-Arg | N-Source | 20418 | Positive |
| PM06 | B05 | Arg-Ala | N-Source | 19944 | Positive |
| PM06 | C03 | Arg-Ser | N-Source | 19853 | Positive |
| PM06 | B08 | Arg-Gln | N-Source | 19811 | Positive |
| PM06 | B09 | Arg-Glu | N-Source | 19168 | Positive |
| PM06 | D05 | Gln-Gln | N-Source | 18204 | Positive |
| PM06 | B07 | Arg-Asp | N-Source | 18021 | Positive |
| PM06 | A12 | Ala-Pro | N-Source | 17699 | Positive |
| PM06 | F03 | Gly-Tyr | N-Source | 17509 | Positive |
| PM06 | F11 | His-Ser | N-Source | 17207 | Positive |
| PM06 | D06 | Gln-Gly | N-Source | 16955 | Positive |
| PM06 | F06 | His-Gly | N-Source | 16933 | Positive |
| PM06 | A06 | Ala-Glu | N-Source | 16796 | Positive |
| PM06 | A08 | Ala-His | N-Source | 16365 | Positive |
| PM06 | G05 | Ile-Gln | N-Source | 16200 | Positive |
| PM06 | A10 | Ala-Lys | N-Source | 16041 | Positive |
| PM06 | A05 | Ala-Asn | N-Source | 16038 | Positive |
| PM06 | G07 | Ile-His | N-Source | 15942 | Positive |
| PM06 | E06 | Gly-His | N-Source | 15698 | Positive |
| PM06 | G01 | His-Tyr | N-Source | 15418 | Positive |
| PM06 | G02 | His-Val | N-Source | 15383 | Positive |
| PM06 | A03 | Ala-Ala | N-Source | 15356 | Positive |
| PM06 | H04 | Leu-Ala | N-Source | 15228 | Positive |
| PM06 | B01 | Ala-Ser | N-Source | 15026 | Positive |
| PM06 | A11 | Ala-Phe | N-Source | 14736 | Positive |
| PM06 | E12 | Gly-Ser | N-Source | 14591 | Positive |
| PM06 | C08 | Asn-Val | N-Source | 14590 | Positive |
| PM06 | E11 | Gly-Pro | N-Source | 14282 | Positive |
| PM06 | C12 | Asp-Lys | N-Source | 13992 | Positive |
| PM06 | B02 | Ala-Thr | N-Source | 13989 | Positive |
| PM06 | G03 | Ile-Ala | N-Source | 13848 | Positive |
| PM06 | A09 | Ala-Leu | N-Source | 13775 | Positive |
| PM06 | F07 | His-Leu | N-Source | 13664 | Positive |
| PM06 | C07 | Asn-Glu | N-Source | 13634 | Positive |
| PM06 | A07 | Ala-Gly | N-Source | 13208 | Positive |
| PM06 | F10 | His-Pro | N-Source | 13122 | Positive |
| PM06 | F05 | His-Asp | N-Source | 12940 | Positive |
| PM06 | B03 | Ala-Trp | N-Source | 12884 | Positive |
| PM06 | G12 | Ile-Ser | N-Source | 12854 | Positive |
| PM06 | E02 | Gly-Ala | N-Source | 12414 | Positive |
| PM06 | F08 | His-Lys | N-Source | 12143 | Positive |
| PM06 | H03 | Ile-Val | N-Source | 12087 | Positive |
| PM06 | E05 | Gly-Gly | N-Source | 12003 | Positive |
| PM06 | G06 | Ile-Gly | N-Source | 11604 | Positive |
| PM06 | F04 | Gly-Val | N-Source | 11259 | Positive |
| PM06 | E07 | Gly-Leu | N-Source | 10834 | Positive |
| PM06 | H06 | Leu-Asp | N-Source | 10753 | Positive |
| PM06 | D03 | Asp-Val | N-Source | 10648 | Positive |
| PM06 | D04 | Cys-Gly | N-Source | 10456 | Positive |
| PM06 | G11 | Ile-Pro | N-Source | 9944 | Marginal |
| PM06 | F01 | Gly-Thr | N-Source | 9883 | Marginal |
| PM06 | F09 | His-Met | N-Source | 9819 | Marginal |
| PM06 | H08 | Leu-Gly | N-Source | 9656 | Marginal |
| PM06 | E01 | Glu-Val | N-Source | 9412 | Marginal |
| PM06 | E08 | Gly-Lys | N-Source | 9248 | Marginal |
| PM06 | H07 | Leu-Glu | N-Source | 9097 | Marginal |
| PM06 | C11 | Asp-Leu | N-Source | 8873 | Marginal |
| PM06 | E10 | Gly-Phe | N-Source | 8871 | Marginal |
| PM06 | D12 | Glu-Tyr | N-Source | 7484 | Marginal |
| PM06 | D09 | Glu-Gly | N-Source | 7386 | Marginal |
| PM06 | D07 | Glu-Asp | N-Source | 6224 | Marginal |
| PM06 | D02 | Asp-Trp | N-Source | 5826 | Marginal |
| PM06 | C09 | Asp-Asp | N-Source | 5240 | Marginal |
| PM06 | F02 | Gly-Trp | N-Source | 5229 | Marginal |
| PM06 | G08 | Ile-Ile | N-Source | 5206 | Marginal |
| PM06 | D08 | Glu-Glu | N-Source | 5043 | Marginal |
| PM06 | F12 | His-Trp | N-Source | 4940 | Marginal |
| PM06 | D01 | Asp-Phe | N-Source | 4903 | Marginal |
| PM06 | G10 | Ile-Phe | N-Source | 4830 | Marginal |
| PM06 | D10 | Glu-Ser | N-Source | 4806 | Marginal |
| PM06 | C10 | Asp-Glu | N-Source | 4667 | Marginal |
| PM06 | H09 | Leu-Ile | N-Source | 4516 | Marginal |
| PM06 | E04 | Gly-Cys | N-Source | 4010 | Marginal |
| PM06 | E09 | Gly-Met | N-Source | 3329 | Marginal |
| PM06 | H12 | Leu-Phe | N-Source | 3012 | Marginal |
| PM06 | D11 | Glu-Trp | N-Source | 1690 | Marginal |
| PM06 | H02 | Ile-Tyr | N-Source | 1249 | Marginal |
| PM06 | G09 | Ile-Met | N-Source | 623 | None |
| PM06 | A01 | Background | N-Source | 0 | None |
| PM06 | H01 | Ile-Trp | N-Source | -150 | None |
| PM06 | H10 | Leu-Leu | N-Source | -918 | None |
| PM06 | H11 | Leu-Met | N-Source | -1853 | None |
| PM07 | H03 | Val-Arg | N-Source | 25975 | Positive |
| PM07 | E12 | Thr-Arg | N-Source | 24644 | Positive |
| PM07 | F07 | Trp-Arg | N-Source | 23100 | Positive |
| PM07 | A02 | Background | N-Source | 22368 | Positive |
| PM07 | A07 | Lys-Arg | N-Source | 21687 | Positive |
| PM07 | G09 | Tyr-His | N-Source | 20171 | Positive |
| PM07 | F12 | Trp-Lys | N-Source | 19947 | Positive |
| PM07 | H07 | Val-His | N-Source | 19905 | Positive |
| PM07 | B07 | Met-Arg | N-Source | 19498 | Positive |
| PM07 | G06 | Tyr-Gln | N-Source | 19189 | Positive |
| PM07 | D12 | Pro-Tyr | N-Source | 18513 | Positive |
| PM07 | H04 | Val-Asp | N-source | 18253 | Positive |
| PM07 | E03 | Ser-His | N-Source | 17960 | Positive |
| PM07 | E07 | Ser-Pro | N-Source | 16882 | Positive |
| PM07 | D06 | Pro-Gln | N-Source | 16634 | Positive |
| PM07 | G01 | Trp-Phe | N-Source | 16236 | Positive |
| PM07 | G08 | Tyr-Gly | N-Source | 15985 | Positive |
| PM07 | G11 | Tyr-Lys | N-Source | 15894 | Positive |
| PM07 | G05 | Tyr-Ala | N-Source | 15710 | Positive |
| PM07 | A03 | Leu-Ser | N-Source | 14887 | Positive |
| PM07 | B01 | Lys-Pro | N-Source | 14031 | Positive |
| PM07 | B12 | Met-His | N-Source | 13970 | Positive |
| PM07 | B02 | Lys-Ser | N-Source | 13902 | Positive |
| PM07 | F06 | Trp-Ala | N-Source | 13604 | Positive |
| PM07 | D01 | Phe-Pro | N-Source | 13593 | Positive |
| PM07 | H06 | Val-Gly | N-Source | 13342 | Positive |
| PM07 | G02 | Trp-Ser | N-Source | 13184 | Positive |
| PM07 | D11 | Pro-Pro | N-Source | 13063 | Positive |
| PM07 | E11 | Thr-Ala | N-Source | 12890 | Positive |
| PM07 | A06 | Lys-Ala | N-Source | 12669 | Positive |
| PM07 | E01 | Ser-Ala | N-Source | 12402 | Positive |
| PM07 | F05 | Thr-Pro | N-Source | 12244 | Positive |
| PM07 | E09 | Ser-Tyr | N-Source | 12233 | Positive |
| PM07 | D07 | Pro-Gly | N-Source | 12168 | Positive |
| PM07 | B05 | Lys-Tyr | N-Source | 12011 | Positive |
| PM07 | A08 | Lys-Glu | N-Source | 11608 | Positive |
| PM07 | H08 | Val-Ile | N-Source | 11397 | Positive |
| PM07 | D04 | Pro-Ala | N-Source | 11358 | Positive |
| PM07 | E02 | Ser-Gly | N-Source | 10958 | Positive |
| PM07 | E10 | Ser-Val | N-Source | 10532 | Positive |
| PM07 | E06 | Ser-Phe | N-Source | 10283 | Positive |
| PM07 | H12 | G-Glu-Gly | N-Source | 10196 | Positive |
| PM07 | H05 | Val-Asp | N-Source | 9951 | Marginal |
| PM07 | F02 | Thr-Gly | N-Source | 9673 | Marginal |
| PM07 | A05 | Leu-Val | N-Source | 9656 | Marginal |
| PM07 | E08 | Ser-Ser | N-Source | 9564 | Marginal |
| PM07 | B06 | Lys-Val | N-Source | 9489 | Marginal |
| PM07 | D10 | Pro-Phe | N-Source | 9376 | Marginal |
| PM07 | B03 | Lys-Thr | N-Source | 9015 | Marginal |
| PM07 | C10 | Phe-Gly | N-Source | 8902 | Marginal |
| PM07 | A12 | Lys-Phe | N-Source | 8899 | Marginal |
| PM07 | E04 | Ser-Leu | N-Source | 8778 | Marginal |
| PM07 | H09 | Val-Leu | N-Source | 8723 | Marginal |
| PM07 | B09 | Met-Gln | N-Source | 8644 | Marginal |
| PM07 | G12 | Tyr-Phe | N-Source | 8615 | Marginal |
| PM07 | D02 | Phe-Ser | N-Source | 8567 | Marginal |
| PM07 | A10 | Lys-Leu | N-Source | 8401 | Marginal |
| PM07 | B04 | Lys-Trp | N-Source | 8234 | Marginal |
| PM07 | G07 | Tyr-Glu | N-Source | 7626 | Marginal |
| PM07 | C09 | Phe-Ala | N-Source | 7510 | Marginal |
| PM07 | H11 | Val-Val | N-Source | 7336 | Marginal |
| PM07 | F10 | Trp-Gly | N-Source | 7227 | Marginal |
| PM07 | F01 | Thr-Glu | N-Source | 6858 | Marginal |
| PM07 | A09 | Lys-Ile | N-Source | 6843 | Marginal |
| PM07 | D09 | Pro-Leu | N-Source | 6774 | Marginal |
| PM07 | A04 | Leu-Trp | N-Source | 6680 | Marginal |
| PM07 | F03 | Thr-Leu | N-Source | 6337 | Marginal |
| PM07 | C06 | Met-Pro | N-Source | 6196 | Marginal |
| PM07 | H02 | Tyr-Tyr | N-Source | 4925 | Marginal |
| PM07 | C11 | Phe-Ile | N-Source | 4851 | Marginal |
| PM07 | G03 | Trp-Trp | N-Source | 4804 | Marginal |
| PM07 | B08 | Met-Asp | N-Source | 4213 | Marginal |
| PM07 | C12 | Phe-Phe | N-Source | 3707 | Marginal |
| PM07 | D05 | Pro-Asp | N-Source | 3605 | Marginal |
| PM07 | G10 | Tyr-Leu | N-Source | 3314 | Marginal |
| PM07 | H10 | Val-Ser | N-source | 3102 | Marginal |
| PM07 | E05 | Ser-Met | N-Source | 2910 | Marginal |
| PM07 | F11 | Trp-Leu | N-Source | 2737 | Marginal |
| PM07 | C03 | Met-Lys | N-Source | 2497 | Marginal |
| PM07 | C08 | Met-Val | N-Source | 2422 | Marginal |
| PM07 | A11 | Lys-Lys | N-Source | 2358 | Marginal |
| PM07 | F04 | Thr-Met | N-Source | 2310 | Marginal |
| PM07 | B11 | Met-Gly | N-Source | 2037 | Marginal |
| PM07 | B10 | Met-Glu | N-Source | 1851 | Marginal |
| PM07 | G04 | Trp-Tyr | N-Source | 1512 | Marginal |
| PM07 | H01 | Tyr-Trp | N-Source | 1289 | Marginal |
| PM07 | C07 | Met-Trp | N-Source | 1026 | Marginal |
| PM07 | C01 | Met-Ile | N-Source | 922 | None |
| PM07 | F08 | Trp-Asp | N-Source | 586 | None |
| PM07 | C02 | Met-Leu | N-Source | 286 | None |
| PM07 | D03 | Phe-Trp | N-Source | 62 | None |
| PM07 | F09 | Trp-Glu | N-Source | 62 | None |
| PM07 | C05 | Met-Phe | N-Source | 31 | None |
| PM07 | A01 | Background | N-Source | 0 | None |
| PM07 | C04 | Met-Met | N-Source | -2 | None |
| PM07 | D08 | Pro-Hyp | N-Source | -2 | None |
| PM08 | A02 | Positive Control | N-Source | 21197 | Positive |
| PM08 | C12 | Pro-Asn | N-Source | 19085 | Positive |
| PM08 | E12 | Val-Pro | N-Source | 18702 | Positive |
| PM08 | D12 | Thr-Gln | N-Source | 18687 | Positive |
| PM08 | A04 | Ala-Gln | N-Source | 18355 | Positive |
| PM08 | B03 | His-Ala | N-Source | 17668 | Positive |
| PM08 | B05 | His-His | N-Source | 17591 | Positive |
| PM08 | C11 | Pro-Arg | N-Source | 17505 | Positive |
| PM08 | H01 | Gly-Gly-Ala | N-Source | 17399 | Positive |
| PM08 | B04 | His-Glu | N-Source | 17197 | Positive |
| PM08 | B09 | Leu-His | N-Source | 17140 | Positive |
| PM08 | G11 | Ala-Ala-Ala | N-Source | 16815 | Positive |
| PM08 | H06 | Gly-Gly-Phe | N-Source | 16798 | Positive |
| PM08 | B06 | Ile-Asn | N-Source | 16764 | Positive |
| PM08 | H04 | Gly-Gly-Ile | N-Source | 16463 | Positive |
| PM08 | D07 | Ser-Asn | N-Source | 16458 | Positive |
| PM08 | B08 | Leu-Asn | N-Source | 16272 | Positive |
| PM08 | F04 | b-Ala-His | N-Source | 16238 | Positive |
| PM08 | A12 | Gly-Asn | N-Source | 16188 | Positive |
| PM08 | H05 | Gly-Gly-Leu | N-Source | 15389 | Positive |
| PM08 | H03 | Gly-Gly-Gly | N-Source | 15319 | Positive |
| PM08 | E07 | Val-Gln | N-Source | 15303 | Positive |
| PM08 | F07 | D-Ala-D-Ala | N-Source | 14909 | Positive |
| PM08 | A03 | Ala-Asp | N-Source | 14781 | Positive |
| PM08 | D09 | Ser-Gln | N-Source | 14690 | Positive |
| PM08 | C07 | Gln-Glu | N-Source | 14045 | Positive |
| PM08 | B11 | Leu-Tyr | N-Source | 13845 | Positive |
| PM08 | H09 | Leu-Gly-Gly | N-Source | 13683 | Positive |
| PM08 | F01 | Val-Ser | N-Source | 13647 | Positive |
| PM08 | G01 | G-Glu-Gly | N-Source | 13476 | Positive |
| PM08 | B12 | Lys-Asp | N-Source | 13416 | Positive |
| PM08 | C01 | Lys-Gly | N-Source | 13250 | Positive |
| PM08 | A09 | Asp-Gln | N-Source | 13005 | Positive |
| PM08 | H08 | Gly-Phe-Phe | N-Source | 12838 | Positive |
| PM08 | A05 | Ala-Ile | N-Source | 12608 | Positive |
| PM08 | H12 | Tyr-Gly-Gly | N-Source | 12468 | Positive |
| PM08 | G03 | Gly-D-Ala | N-Source | 12309 | Positive |
| PM08 | A11 | Glu-Ala | N-Source | 12099 | Positive |
| PM08 | B02 | Gly-Ile | N-Source | 12046 | Positive |
| PM08 | A08 | Asp-Ala | N-Source | 11699 | Positive |
| PM08 | B10 | Leu-Pro | N-Source | 11512 | Positive |
| PM08 | A07 | Ala-Val | N-Source | 11506 | Positive |
| PM08 | E05 | Tyr-Val | N-Source | 11294 | Positive |
| PM08 | D06 | Pro-Val | N-Source | 11238 | Positive |
| PM08 | D01 | Pro-Glu | N-Source | 11039 | Positive |
| PM08 | C10 | Phe-Val | N-Source | 11026 | Positive |
| PM08 | D04 | Pro-Ser | N-Source | 10920 | Positive |
| PM08 | D10 | Ser-Glu | N-Source | 10435 | Positive |
| PM08 | D02 | Pro-lle | N-Source | 10354 | Positive |
| PM08 | E09 | Val-Lys | N-Source | 10181 | Positive |
| PM08 | F08 | D-Ala-Gly | N-Source | 10022 | Positive |
| PM08 | G08 | Leu-B-Ala | N-Source | 10003 | Positive |
| PM08 | F02 | b-Ala-Ala | N-Source | 9461 | Marginal |
| PM08 | E06 | Val-Ala | N-Source | 9425 | Marginal |
| PM08 | D03 | Pro-Lys | N-Source | 9400 | Marginal |
| PM08 | E01 | Thr-Phe | N-Source | 9343 | Marginal |
| PM08 | A06 | Ala-Met | N-Source | 9261 | Marginal |
| PM08 | E02 | Thr-Ser | N-Source | 9180 | Marginal |
| PM08 | F03 | B-Ala-Gly | N-Source | 8857 | Marginal |
| PM08 | E03 | Trp-Val | N-Source | 8839 | Marginal |
| PM08 | B07 | Ile-Leu | N-Source | 8730 | Marginal |
| PM08 | G10 | Phe-B-Ala | N-Source | 8669 | Marginal |
| PM08 | B01 | Glycyl-L-Aspartic Acid | N-Source | 7832 | Marginal |
| PM08 | E08 | Val-Glu | N-Source | 7285 | Marginal |
| PM08 | D08 | Ser-Asp | N-Source | 7196 | Marginal |
| PM08 | C06 | Phe-Glu | N-Source | 6865 | Marginal |
| PM08 | E04 | Tyr-Ile | N-Source | 6503 | Marginal |
| PM08 | C05 | Phe-Asp | N-Source | 6266 | Marginal |
| PM08 | D11 | Thr-Asp | N-Source | 5296 | Marginal |
| PM08 | E11 | Val-Phe | N-Source | 5280 | Marginal |
| PM08 | H11 | Phe- Gly-Gly | N-Source | 5189 | Marginal |
| PM08 | C02 | Lys-Met | N-Source | 4809 | Marginal |
| PM08 | A10 | Asp-Gly | N-Source | 4740 | Marginal |
| PM08 | C03 | Met-Thr | N-Source | 4658 | Marginal |
| PM08 | C04 | Met-Tyr | N-Source | 3918 | Marginal |
| PM08 | F05 | Met-b-Ala | N-Source | 3447 | Marginal |
| PM08 | E10 | Val-Met | N-Source | 3141 | Marginal |
| PM08 | H07 | Val-Tyr-Val | N-Source | 2542 | Marginal |
| PM08 | F09 | D-Ala-Leu | N-Source | 2121 | Marginal |
| PM08 | C08 | Phe-Met | N-Source | 1486 | Marginal |
| PM08 | G05 | Gly-D-Ser | N-Source | 1319 | Marginal |
| PM08 | C09 | Phe-Tyr | N-Source | 1217 | Marginal |
| PM08 | F06 | b-Ala-Phe | N-Source | 753 | None |
| PM08 | H10 | Leu-Leu-Leu | N-Source | 737 | None |
| PM08 | G12 | Ala-Gly-Gly (D-) | N-Source | 448 | None |
| PM08 | H02 | Gly-Gly-D-Leu | N-Source | 25 | None |
| PM08 | G02 | G-D-Glu-Gly | N-Source | 11 | None |
| PM08 | A01 | Background | N-Source | 0 | None |
| PM08 | D05 | Pro-Trp | N-Source | -72 | None |
| PM08 | F12 | D-Leu-Tyr | N-Source | -81 | None |
| PM08 | G07 | Gly-D-Val | N-Source | -85 | None |
| PM08 | G04 | Gly-D-Asp | N-Source | -107 | None |
| PM08 | G06 | Gly-D-Thr | N-Source | -113 | None |
| PM08 | F10 | D-leu-D-Leu | N-Source | -120 | None |
| PM08 | F11 | D-Leu-Gly | N-Source | -120 | None |
| PM08 | G09 | Leu-D-Leu | N-Source | -120 | None |

^a^Colourimetric change in arbitrary units for substrate oxidation based on the area under the pseudokinetic curve, with the value of the background control subtracted.
