# Supplementary material for: High-Throughput Phenotypic Characterization of Pseudomonas aeruginosa Membrane Transport Genes
Source: PLoS Genet. 2008 Oct 3;4(10):e1000211. doi: 10.1371/journal.pgen.1000211 (PMC2542419; doi:10.1371/journal.pgen.1000211)
Supplement: Table S3 — Strain list. (0.49 MB DOC) [file pgen.1000211.s003.doc]

**Table S3**. Strain list.

| ***host strain*** | ***Mutant*** | ***Pa_ID*** | ***Function*** | ***Gene name*** |
| --- | --- | --- | --- | --- |
| PAK | 001F08 | 4707 | probable permease of ABC transporter |  |
| PAK | 001H08 | 3467 | probable MFS transporter |  |
| PAK | 002A01 | 3264 | probable transporter |  |
| PAK | 003E06 | 4825 | Mg(2+) transport ATPase, P-type 2 | mgtA |
| PAK | 004F08 | 137 | probable permease of ABC transporter |  |
| PAK | 007H01 | 1051 | probable transporter |  |
| PAK | 008B09 | 1386 | probable ATP-binding component of ABC transporter |  |
| PAK | 008B10 | 3858 | probable amino acid-binding protein |  |
| PAK | 008C05 | 3677 | probable RND efflux membrane fusion protein precursor |  |
| PAK | 009-D04 | 917 | potassium uptake protein Kup | kup |
| PAK | 009H01 | 3641 | probable amino acid permease |  |
| PAK | 010A08 | 304 | polyamine transport protein PotI | potI |
| PAK | 010D04 | 5170 | arginine/ornithine antiporter | arcD |
| PAK | 010H06 | 3672 | probable ATP-binding component of ABC transporter |  |
| PAK | 011A02 | 2563 | probable sulfate transporter |  |
| PAK | 011A09 | 4206 | probable RND efflux membrane fusion protein precursor |  |
| PAK | 011E08 | 1286 | probable MFS transporter |  |
| PAK | 012G09 | 322 | probable transporter |  |
| PAK | 014E02 | 5168 | probable dicarboxylate transporter |  |
| PAK | 014E12 | 157 | probable RND efflux membrane fusion protein precursor |  |
| PAK | 015A10 | 5479 | proton-glutamate symporter | gltP |
| PAK | 015F10 | 1993 | probable MFS transporter |  |
| PAK | 015G03 | 4594 | probable ATP-binding component of ABC transporter |  |
| PAK | 015H10 | 5230 | probable permease of ABC transporter |  |
| PAK | 016A05 | 2114 | probable MFS transporter |  |
| PAK | 016B11 | 2408 | probable ATP-binding component of ABC transporter |  |
| PAK | 016D01 | 4910 | probable ATP-binding component of ABC transporter |  |
| PAK | 017D11 | 1808 | probable permease of ABC transporter |  |
| PAK | 017E05 | 5094 | probable ATP-binding component of ABC transporter |  |
| PAK | 017G06 | 3188 | probable permease of ABC sugar transporter |  |
| PAK | 021E01 | 3865 | probable amino acid binding protein |  |
| PAK | 021F09 | 4355 | probable MFS transporter |  |
| PAK | 023A06 | 3963 | probable transporter |  |
| PAK | 023B07 | 4072 | probable amino acid permease |  |
| PAK | 023C09 | 4497 | probable binding protein component of ABC transporter |  |
| PAK | 023C11 | 4193 | probable permease of ABC transporter |  |
| PAK | 023D11 | 3441 | probable molybdopterin-binding protein |  |
| PAK | 024A04 | 3760 | probable phosphotransferase protein |  |
| PAK | 025A03 | 2341 | probable ATP-binding component of ABC maltose/mannitol transporter |  |
| PAK | 025C02 | 2925 | histidine transport system permease HisM | hisM |
| PAK | 025D06 | 4506 | probable ATP-binding component of ABC dipeptide transporter |  |
| PAK | 026B08 | 215 | probable transporter |  |
| PAK | 026E02 | 4804 | probable amino acid permease |  |
| PAK | 027D01 | 3920 | probable metal transporting P-type ATPase |  |
| PAK | 027F02 | 605 | probable permease of ABC transporter |  |
| PAK | 027G12 | 1634 | potassium-transporting ATPase, B chain | kdpB |
| PAK | 028B08 | 4223 | probable ATP-binding component of ABC transporter |  |
| PAK | 028H01 | 3039 | probable transporter |  |
| PAK | 029C02 | 1194 | probable amino acid permease |  |
| PAK | 029G11 | 1073 | branched-chain amino acid transport protein BraD | braD |
| PAK | 030B11 | 2314 | probable MFS transporter |  |
| PAK | 030E04 | 2912 | probable ATP-binding component of ABC transporter |  |
| PAK | 030H05 | 1297 | probable metal transporter |  |
| PAK | 031B07 | 2526 | probable RND efflux transporter |  |
| PAK | 031C10 | 3901 | Fe(III) dicitrate transport protein FecA | fecA |
| PAK | 031E09 | 326 | probable ATP-binding component of ABC transporter |  |
| PAK | 031F08 | 3448 | probable permease of ABC transporter |  |
| PAK | 032B02 | 604 | probable binding protein component of ABC transporter |  |
| PAK | 032C09 | 3382 | phosphonate transport protein PhnE | phnE |
| PAK | 033A05 | 1861 | molybdenum transport protein ModC | modC |
| PAK | 033E02 | 3538 | probable ATP-binding component of ABC transporter |  |
| PAK | 033F07 | 2923 | periplasmic histidine-binding protein HisJ | hisJ |
| PAK | 033G03 | 5367 | membrane protein component of ABC phosphate transporter | pstA |
| PAK | 033G11 | 4343 | probable MFS transporter |  |
| PAK | 034B09 | 4628 | lysine-specific permease | lysP |
| PAK | 035B06 | 5169 | probable C4-dicarboxylate transporter |  |
| PAK | 035D12 | 3607 | polyamine transport protein PotA | potA |
| PAK | 035F04 | 438 | cytosine permease | codB |
| PAK | 035H01 | 4821 | probable transporter |  |
| PAK | 037A06 | 2135 | probable transporter |  |
| PAK | 037F03 | 2202 | probable amino acid permease |  |
| PAK | 038F07 | 450 | probable phosphate transporter |  |
| PAK | 039A10 | 2701 | probable MFS transporter |  |
| PAK | 039B12 | 886 | probable C4-dicarboxylate transporter |  |
| PAK | 040A02 | 303 | polyamine transport protein PotH | potH |
| PAK | 040D05 | 4207 | probable RND efflux transporter |  |
| PAK | 041D07 | 892 | arginine/ornithine transport protein AotP | aotP |
| PAK | 041H09 | 220 | probable amino acid permease |  |
| PAK | 042C10 | 884 | probable C4-dicarboxylate-binding periplasmic protein |  |
| PAK | 043C05 | 5217 | probable binding protein component of ABC iron transporter |  |
| PAK | 043D05 | 3236 | probable glycine betaine-binding protein precursor |  |
| PAK | 043E06 | 1019 | cis,cis-muconate transporter MucK | mucK |
| PAK | 043G08 | 1436 | probable RND efflux transporter |  |
| PAK | 044C05 | 2812 | probable ATP-binding component of ABC transporter |  |
| PAK | 044C11 | 2307 | probable permease of ABC transporter |  |
| PAK | 044D09 | 158 | probable RND efflux transporter |  |
| PAK | 044E09 | 3937 | probable ATP-binding component of ABC taurine transporter |  |
| PAK | 044F07 | 3749 | probable MFS transporter |  |
| PAK | 044F11 | 1147 | probable amino acid permease |  |
| PAK | 045C02 | 1916 | probable amino acid permease |  |
| PAK | 045D06 | 1144 | probable MFS transporter |  |
| PAK | 047A10 | 5510 | probable transporter |  |
| PAK | 047C02 | 5153 | probable periplasmic binding protein |  |
| PAK | 047C04 | 5375 | choline transporter BetT | betT1 |
| PAK | 047E10 | 3889 | probable binding protein component of ABC transporter |  |
| PAK | 049C04 | 3210 | potassium uptake protein TrkH | trkH |
| PAK | 049F03 | 2472 | probable MFS transporter |  |
| PAK | 050B04 | 2203 | probable amino acid permease |  |
| PAK | 050G07 | 2068 | probable MFS transporter |  |
| PAK | 051D06 | 4913 | probable binding protein component of ABC transporter |  |
| PAK | 051D12 | 5030 | probable MFS transporter |  |
| PAK | 051E12 | 129 | gamma-aminobutyrate permease | gabP |
| PAK | 051F03 | 313 | probable permease of ABC transporter |  |
| PAK | 052D06 | 4158 | ferric enterobactin transport protein FepC | fepC |
| PAK | 052D11 | 3375 | probable ATP-binding component of ABC transporter |  |
| PAK | 052F02 | 1282 | probable MFS transporter |  |
| PAK | 052F06 | 235 | 4-hydroxybenzoate transporter PcaK | pcaK |
| PAK | 052G01 | 5501 | permease of ABC zinc transporter ZnuB | znuB |
| PAK | 052G04 | 3000 | aromatic amino acid transport protein AroP1 | aroP1 |
| PAK | 053A01 | 3761 | probable phosphotransferase system protein |  |
| PAK | 053B10 | 2528 | probable RND efflux membrane fusion protein precursor |  |
| PAK | 053F06 | 5311 | probable MFS transporter |  |
| PAK | 054B06 | 1418 | probable sodium:solute symport protein |  |
| PAK | 054C05 | 1491 | probable transporter |  |
| PAK | 054C09 | 846 | probable sulfate uptake protein |  |
| PAK | 055B02 | 2041 | probable amino acid permease |  |
| PAK | 055E01 | 2592 | probable periplasmic spermidine/putrescine-binding protein |  |
| PAK | 055G06 | 3595 | probable MFS transporter |  |
| PAK | 055G12 | 603 | probable ATP-binding component of ABC transporter |  |
| PAK | 056E10 | 5434 | tryptophan permease | mtr |
| PAK | 057B09 | 16 | potassium uptake protein TrkA | trkA |
| PAK | 057E11 | 5530 | probable MFS dicarboxylate transporter |  |
| PAK | 057H01 | 3560 | phosphotransferase system, fructose-specific IIBC component | fruA |
| PAK | 058B05 | 2924 | histidine transport system permease HisQ | hisQ |
| PAK | 058C01 | 4096 | probable MFS transporter |  |
| PAK | 059F09 | 4365 | probable transporter |  |
| PAK | 059H08 | 4195 | probable binding protein component of ABC transporter |  |
| PAK | 060E04 | 5216 | probable permease of ABC iron transporter |  |
| PAK pili- | 061E12 | 3610 | polyamine transport protein PotD | potD |
| PAK pili- | 062B02 | 888 | arginine/ornithine binding protein AotJ | aotJ |
| PAK | 063G04 | 3253 | probable permease of ABC transporter |  |
| PAK | 064F02 | 1682 | probable MFS metabolite transporter |  |
| PAK | 064F08 | 2339 | probable binding-protein-dependent maltose/mannitol transport protein |  |
| PAK | 064G02 | 136 | probable ATP-binding component of ABC transporter |  |
| PAK | 064G06 | 2520 | RND divalent metal cation efflux transporter CzcA | czcA |
| PAK pili- | 065A01 | 186 | probable binding protein component of ABC transporter |  |
| PAK pili- | 065D12 | 5368 | membrane protein component of ABC phosphate transporter | pstC |
| PAK | 066D04 | 206 | probable ATP-binding component of ABC transporter |  |
| PAK | 066E09 | 4912 | probable permease of ABC branched chain amino acid transporter |  |
| PAK pili- | 068F11 | 1258 | probable permease of ABC transporter |  |
| PAK pili- | 069B08 | 3839 | probable sodium:sulfate symporter |  |
| PAK pili- | 069H02 | 1947 | ribose transport protein RbsA | rbsA |
| PAK pili- | 069H03 | 1590 | branched chain amino acid transporter | braB |
| PAK pili- | 070A04 | 5155 | probable permease of ABC transporter |  |
| PAK pili- | 070A10 | 1569 | probable MFS transporter |  |
| PAK pili- | 070B03 | 5252 | probable ATP-binding component of ABC transporter |  |
| PAK pili- | 070E05 | 1519 | probable transporter |  |
| PAK pili- | 070F09 | 5160 | drug efflux transporter |  |
| PAK pili- | 071E07 | 2340 | probable binding-protein-dependent maltose/mannitol transport protein |  |
| PAK pili- | 071G01 | 4334 | probable transport protein |  |
| PAK | 072A11 | 3394 | NosF protein | nosF |
| PAK | 072D09 | 2711 | probable periplasmic spermidine/putrescine-binding protein |  |
| PAK | 072D11 | 1819 | probable amino acid permease |  |
| PAK | 072E08 | 4903 | probable MFS transporter |  |
| PAK | 072E11 | 325 | probable permease of ABC transporter |  |
| PAK | 072G09 | 703 | probable MFS transporter |  |
| PAK | 072G11 | 1958 | probable transporter |  |
| PAK | 072H05 | 1626 | probable MFS transporter |  |
| PAK | 073B06 | 3887 | Na+/H+ antiporter NhaP | nhaP |
| PAK | 073D10 | 302 | polyamine transport protein PotG | potG |
| PAK | 074F03 | 4719 | probable transporter |  |
| PAK | 074G11 | 4126 | probable MFS transporter |  |
| PAK | 076B02 | 5235 | glycerol-3-phosphate transporter | glpT |
| PAK | 076C01 | 1419 | probable transporter |  |
| PAK | 079G07 | 3316 | probable permease of ABC transporter |  |
| PAK | 079H01 | 1108 | probable MFS transporter |  |
| PAK | 080F10 | 3447 | probable ATP-binding component of ABC transporter |  |
| PAK | 081A03 | 1650 | probable transporter |  |
| PAK | 081D11 | 4647 | uracil permease | uraA |
| PAK | 081E12 | 3718 | probable MFS transporter |  |
| PAK | 082F09 | 1410 | probable periplasmic spermidine/putrescine-binding protein |  |
| PAK | 082F10 | 5207 | probable phosphate transporter |  |
| PAK | 082H10 | 73 | probable ATP-binding component of ABC transporter |  |
| PAK | 083F08 | 3254 | probable ATP-binding component of ABC transporter |  |
| PAK | 083F09 | 2435 | probable cation-transporting P-type ATPase |  |
| PAK | 084A11 | 4614 | conductance mechanosensitive channel | mscL |
| PAK | 084B01 | 1908 | probable MFS transporter |  |
| PAK | 084B10 | 866 | aromatic amino acid transport protein AroP2 | aroP2 |
| PAK | 084C04 | 4770 | L-lactate permease | lldP |
| PAK | 084C05 | 2055 | probable MFS transporter |  |
| PAK | 084D04 | 2938 | probable transporter |  |
| PAK | 084F12 | 156 | probable RND efflux membrane fusion protein precursor |  |
| PAK | 085B09 | 5518 | probable potassium efflux transporter |  |
| PAK | 085B12 | 3336 | probable MFS transporter |  |
| PAK | 085D10 | 323 | probable binding protein component of ABC transporter |  |
| PAK | 086B09 | 4859 | probable permease of ABC transporter |  |
| PAK | 086C02 | 2058 | probable binding protein component of ABC transporter |  |
| PAK | 086D11 | 246 | probable MFS transporter |  |
| PAK | 086E06 | 783 | sodium/proline symporter PutP | putP |
| PAK | 086E10 | 4143 | probable toxin transporter |  |
| PAK | 086G01 | 1313 | probable MFS transporter |  |
| PAK | 087G03 | 3739 | probable sodium/hydrogen antiporter |  |
| PAK | 088B11 | 3383 | binding protein component of ABC phosphonate transporter |  |
| PAK | 088D10 | 2327 | probable permease of ABC transporter |  |
| PAK | 088D11 | 4194 | probable permease of ABC transporter |  |
| PAK | 088E09 | 3303 | probable MFS transporter |  |
| PAK | 088F07 | 103 | probable sulfate transporter |  |
| PAK | 089B06 | 2210 | probable MFS transporter |  |
| PAK | 090C03 | 5377 | probable permease of ABC transporter |  |
| PAK | 090C11 | 3212 | probable ATP-binding component of ABC transporter |  |
| PAK | 091F07 | 185 | probable permease of ABC transporter |  |
| PAK | 093D02 | 789 | probable amino acid permease |  |
| PAK | 094B05 | 3234 | probable sodium:solute symporter |  |
| PAK | 094G08 | 4113 | probable MFS transporter |  |
| PAK | 096B02 | 3709 | probable MFS transporter |  |
| PAK | 096C02 | 5468 | probable citrate transporter |  |
| PAK | 096C06 | 1429 | probable cation-transporting P-type ATPase |  |
| PAK | 096D03 | 1212 | probable MFS transporter |  |
| PAK | 096D05 | 334 | probable MFS transporter |  |
| PAK | 097B03 | 5167 | probable c4-dicarboxylate-binding protein |  |
| PAK | 097E02 | 5282 | probable MFS transporter |  |
| PAK | 097E03 | 1341 | probable permease of ABC transporter |  |
| PAK | 097G05 | 606 | probable permease of ABC transporter |  |
| PAK | 097G07 | 4187 | probable MFS transporter |  |
| PAK | 102A12 | 4900 | probable MFS transporter |  |
| PAK | 105A07 | 138 | probable permease of ABC transporter |  |
| PAK | 105C08 | 1113 | probable ATP-binding/permease fusion ABC transporter |  |
| PAK | 105F07 | 3938 | probable periplasmic taurine-binding protein precursor |  |
| PAK | 107A02 | 3562 | probable phosphotransferase system enzyme I |  |
| PAK | 107F09 | 3189 | probable permease of ABC sugar transporter |  |
| PAK | 108H03 | 3690 | probable metal-transporting P-type ATPase |  |
| PAK | 108H06 | 5476 | citrate transporter | citA |
| PAK | 109D02 | 5097 | probable amino acid permease |  |
| PAK | 109G08 | 890 | arginine/ornithine transport protein AotM | aotM |
| PAK | 110H06 | 1946 | binding protein component precursor of ABC ribose transporter | rbsB |
| PAK | 111C08 | 2914 | probable permease of ABC transporter |  |
| PAK | 111E12 | 1256 | probable ATP-binding component of ABC transporter |  |
| PAK | 111F12 | 4593 | probable permease of ABC transporter |  |
| PAK | 112H02 | 2322 | gluconate permease |  |
| PAK | 112H11 | 352 | probable transporter |  |
| PAK | 113A02 | 5074 | probable ATP-binding component of ABC transporter |  |
| PAK | 113A04 | 4222 | probable ATP-binding component of ABC transporter |  |
| PAK | 113C05 | 166 | probable transporter |  |
| PAK | 113H10 | 4160 | ferric enterobactin transport protein FepD | fepD |
| PAK | 114B02 | 3019 | probable ATP-binding component of ABC transporter |  |
| PAK | 114B08 | 283 | sulfate-binding protein precursor | sbp |
| PAK | 114C10 | 4375 | probable RND efflux transporter |  |
| PAK | 116A04 | 3597 | probable amino acid permease |  |
| PAK | 116E09 | 2857 | probable ATP-binding component of ABC transporter |  |
| PAK | 117E03 | 4192 | probable ATP-binding component of ABC transporter |  |
| PAK | 118C09 | 4358 | probable ferrous iron transport protein |  |
| PAK | 118D11 | 1072 | branched-chain amino acid transport protein BraE | braE |
| PAK | 118H10 | 4136 | probable MFS transporter |  |
| PAK | 119B09 | 1435 | probable RND efflux membrane fusion protein precursor |  |
| PAK | 119E02 | 2390 | probable ATP-binding/permease fusion ABC transporter |  |
| PAK | 119E12 | 337 | phosphoenolpyruvate-protein phosphotransferase PtsP | ptsP |
| PAK | 120B04 | 2006 | probable MFS transporter |  |
| PAK | 120E02 | 3190 | probable binding protein component of ABC sugar transporter |  |
| PAK | 127C05 | 885 | probable C4-dicarboxylate transporter |  |
| PAK | 127H06 | 5076 | probable binding protein component of ABC transporter |  |
| PAK | 128A07 | 1876 | probable ATP-binding/permease fusion ABC transporter |  |
| PAK | 128A12 | 3676 | probable RND efflux transporter |  |
| PAK | 128B09 | 1070 | branched-chain amino acid transport protein BraG | braG |
| PAK | 128C04 | 3781 | probable transporter |  |
| PAK | 129A09 | 1848 | probable MFS transporter |  |
| PAK | 129C02 | 809 | probable transporter |  |
| PAK | 129G08 | 3376 | probable ATP-binding component of ABC transporter |  |
| PAK | 129G12 | 3573 | probable MFS transporter |  |
| PAK | 129H06 | 2308 | probable ATP-binding component of ABC transporter |  |
| PAK | 130B11 | 3933 | probable choline transporter |  |
| PAK | 132B06 | 1361 | probable transporter |  |
| PAK | 132B11 | 1074 | branched-chain amino acid transport protein BraC | braC |
| PAK | 132E04 | 4687 | ferric iron-binding periplasmic protein HitA | hitA |
| PAK | 133D04 | 2533 | probable sodium:alanine symporter |  |
| PAK | 135H10 | 5287 | ammonium transporter AmtB | amtB |
| PAK | 136H04 | 3228 | probable ATP-binding/permease fusion ABC transporter |  |
| PAK | 137B02 | 1071 | branched-chain amino acid transport protein BraF | braF |
| PAK | 137E08 | 1339 | probable ATP-binding component of ABC transporter |  |
| PAK | 138B02 | 1260 | probable binding protein component of ABC transporter |  |
| PAK | 139F02 | 229 | dicarboxylic acid transporter PcaT | pcaT |
| PAK | 139F09 | 3265 | probable transporter |  |
| PAK | 140B01 | 3837 | probable permease of ABC transporter |  |
| PAK | 140F01 | 3137 | probable MFS transporter |  |
| PAK | 141C08 | 5268 | magnesium/cobalt transport protein | corA |
| PAK | 143B10 | 2835 | probable MFS transporter |  |
| PAK | 144C09 | 4654 | probable MFS transporter |  |
| PAK | 145G08 | 4161 | ferric enterobactin transport protein FepG | fepG |
| PAK | 146H09 | 4218 | probable transporter |  |
| PAK | 147A09 | 3252 | probable permease of ABC transporter |  |
| PAK | 147E03 | 2653 | probable transporter |  |
| PAK | 147G10 | 4860 | probable permease of ABC transporter |  |
| PAK | 148A12 | 2558 | probable transport protein |  |
| PAK | 148B07 | 4023 | probable transport protein |  |
| PAK | 148D04 | 1340 | probable permease of ABC transporter |  |
| PAK | 148G03 | 476 | probable permease |  |
| PAK | 150A06 | 314 | probable binding protein component of ABC transporter |  |
| PA01 | 151G11 | 5082 | probable binding protein component of ABC transporter |  |
| PA01 | 157B03 | 2079 | probable amino acid permease |  |
| PA01 | 157E10 | 1131 | probable MFS transporter |  |
| PA01 | 158B11 | 5075 | probable permease of ABC transporter |  |
| PA01 | 158E10 | 1863 | molybdate-binding periplasmic protein precursor ModA | modA |
| PA01 | 158F08 | 5376 | probable ATP-binding component of ABC transporter |  |
| PA01 | 158G06 | 1809 | probable permease of ABC transporter |  |
| PA01 | 159A08 | 1964 | probable ATP-binding component of ABC transporter |  |
| PA01 | 159C12 | 811 | probable MFS transporter |  |
| PA01 | 159F01 | 4688 | iron (III)-transport system permease HitB | hitB |
| PA01 | 159G06 | 1507 | probable transporter |  |
| PA01 | 160A12 | 2252 | probable AGCS sodium/alanine/glycine symporter |  |
| PA01 | 160F06 | 1807 | probable ATP-binding component of ABC transporter |  |
| PA01 | 160H02 | 397 | probable cation efflux system protein |  |
| PA01 | 161D11 | 1236 | probable MFS transporter |  |
| PA01 | 161H04 | 1549 | probable cation-transporting P-type ATPase |  |
| PA01 | 163G04 | 3608 | polyamine transport protein PotB | potB |
| PA01 | 163H04 | 4504 | probable permease of ABC transporter |  |
| PA01 | 164A08 | 1971 | branched chain amino acid transporter BraZ | braZ |
| PA01 | 167B05 | 4862 | probable ATP-binding component of ABC transporter |  |
| PA01 | 167D10 | 3838 | probable ATP-binding component of ABC transporter |  |
| PA01 | 167H01 | 1497 | probable transporter |  |
| PA01 | 169A04 | 3926 | probable MFS transporter |  |
| PA01 | 170B02 | 281 | sulfate transport protein CysW | cysW |
| PA01 | 170C03 | 282 | sulfate transport protein CysT | cysT |
| PA01 | 172F03 | 2527 | probable RND efflux transporter |  |
| PA01 | 172F08 | 5500 | zinc transport protein ZnuC | znuC |
| PA01 | 172F09 | 4981 | probable amino acid permease |  |
| PA01 | 172G01 | 860 | probable ATP-binding/permease fusion ABC transporter |  |
| PA01 | 172G10 | 295 | probable periplasmic polyamine binding protein |  |
| PA01 | 174B02 | 300 | polyamine transport protein | potF2 |
| PA01 | 174E08 | 5504 | probable permease of ABC transporter |  |
| PA01 | 174F05 | 4622 | probable MFS transporter |  |
| PA01 | 175G02 | 3660 | probable sodium/hydrogen antiporter |  |
| PA01 | 176F01 | 4233 | probable MFS transporter |  |
| PA01 | 176H06 | 2061 | probable ATP-binding component of ABC transporter |  |
| PA01 | 177H08 | 1207 | glutathione-regulated potassium-efflux system protein KefB | kefB |
| PA01 | 178B11 | 4616 | probable c4-dicarboxylate-binding protein |  |
| PA01 | 182B07 | 3187 | probable ATP-binding component of ABC transporter |  |
| PA01 | 182B12 | 241 | probable MFS transporter |  |
| PA01 | 182G12 | 3176 | sodium/glutamate symporter GltS | gltS |
| PA01 | 184B12 | 4496 | probable binding protein component of ABC transporter |  |
| PA01 | 185A05 | 4289 | probable transporter |  |
| PA01 | 185F08 | 5548 | probable MFS transporter |  |
| PA01 | 188B08 | 5021 | probable sodium/hydrogen antiporter |  |
| PA01 | 188C04 | 458 | probable MFS transporter |  |
| PA01 | 188C09 | 5099 | probable transporter |  |
| PA01 | 188D04 | 4909 | probable ATP-binding component of ABC transporter |  |
| PA01 | 188H02 | 1257 | probable permease of ABC transporter |  |
| PA01 | 189G01 | 4503 | probable permease of ABC transporter |  |
| PA01 | 190E04 | 3877 | nitrite extrusion protein 1 | narK1 |
| PA01 | 191C10 | 4505 | probable ATP-binding component of ABC transporter |  |
| PA01 | 191E04 | 3406 | transport protein HasD | hasD |
| PA01 | 191G03 | 5291 | probable choline transporter |  |
| PA01 | 192A06 | 1493 | sulfate-binding protein of ABC transporter | cysP |
| PA01 | 192A12 | 5231 | probable ATP-binding/permease fusion ABC transporter |  |
| PA01 | 193-C10 | 1811 | probable solute-binding protein |  |
| PA01 | 193-F01 | 602 | probable binding protein component of ABC transporter |  |
| PA02 | 193F05 | 3581 | glycerol uptake facilitator protein | glpF |
| PA01 | 195-A08 | 287 | probable sodium:solute symporter |  |
| PA01 | 195-G07 | 4911 | probable permease of ABC branched-chain amino acid transporter |  |
| PA01 | 196-E02 | 3522 | probable RND efflux transporter |  |
| PA01 | 196-F07 | 29 | probable sulfate transporter |  |
| PA01 | 196-H08 | 5096 | probable binding protein component of ABC transporter |  |
| PA01 | 197-B09 | 4159 | ferrienterobactin-binding periplasmic protein precursor FepB | fepB |
| PA01 | 199-E05 | 1262 | probable MFS transporter |  |
| PA01 | 200-A08 | 1485 | probable amino acid permease |  |
| PA01 | 200-H01 | 2204 | probable binding protein component of ABC transporter |  |
| PA01 | 202-C10 | 2933 | probable MFS transporter |  |
| PA01 | 203-A09 | 889 | arginine/ornithine transport protein AotQ | aotQ |
| PA01 | 205-B09 | 4502 | probable binding protein component of ABC transporter |  |
| PA01 | 205-E07 | 2059 | probable permease of ABC transporter |  |
| PA01 | 206-D07 | 1948 | membrane protein component of ABC ribose transporter | rbsC |
| PA01 | 208-D02 | 1316 | probable MFS transporter |  |
| PA01 | 208-H02 | 1651 | probable transporter |  |
| PA01 | 208-H09 | 5370 | probable MFS transporter |  |
| PA01 | 209-D07 | 2262 | probable 2-ketogluconate transporter |  |
| PA01 | 209-D12 | 324 | probable permease of ABC transporter |  |
| PA01 | 211-B02 | 1425 | probable ATP-binding component of ABC transporter |  |
| PA01 | 211-D06 | 5095 | probable permease of ABC transporter |  |
| PA01 | 211-E02 | 4393 | probable permease |  |
| PA01 | 211-F05 | 4500 | probable binding protein component of ABC transporter |  |
| PA01 | 211-H07 | 2278 | ArsB protein | arsB |
| PA01 | 212-E10 | 3766 | probable aromatic amino acid transporter |  |
| PA01 | 213-A08 | 1633 | potassium-transporting ATPase, A chain | kdpA |
| PA01 | 215-E03 | 3514 | probable ATP-binding component of ABC transporter |  |
| PA01 | 218-F11 | 4595 | probable ATP-binding component of ABC transporter |  |
| PA01 | 223-C05 | 3442 | probable ATP-binding component of ABC transporter |  |
| PA01 | 223-E03 | 301 | polyamine transport protein | potF3 |
| PA01 | 223-F11 | 2409 | probable permease of ABC transporter |  |
| PAK | yq004 | 4292 | probable phosphate transporter |  |
| PAK | yq124 | 4374 | probable RND efflux membrane fusion protein precursor |  |
| PAK | yq141 | 3581 | glycerol uptake facilitator protein | glpF |
| PAK | yq258 | 1647 | probable sulfate transporter |  |
| PAK | yq468 | 5366 | ATP-binding component of ABC phosphate transporter | pstB |
| PAK | yq503 | 5070 | transport protein TatC | tatC |
| PAK | yq545 | 2926 | histidine transport protein HisP | hisP |
| PAK | yq572 | 4456 | probable ATP-binding component of ABC transporter |  |
